# Association of glucagon-like peptide-1 receptor agonists with cardiovascular and kidney outcomes in type 2 diabetic kidney transplant recipients

**DOI:** 10.1186/s12933-025-02649-0

**Published:** 2025-02-21

**Authors:** Li-Chun Lin, Jui-Yi Chen, Thomas Tao-Min Huang, Vin-Cent Wu

**Affiliations:** 1 Division of Nephrology, Department of Internal Medicine, National Taiwan University Hospital, Taipei, Taiwan; 2 Division of Nephrology, Department of Internal Medicine, Chi-Mei Medical Center, Tainan, Taiwan; 3 Department of Health and Nutrition, Chia Nan University of Pharmacy and Science, Tainan, Taiwan; 4 Division of Nephrology, Primary Aldosteronism Center of Internal Medicine, National Taiwan University Hospital, Taipei, Taiwan; 5 NSARF (National Taiwan University Hospital Study Group of ARF), and CAKS (Taiwan Consortium for Acute Kidney Injury and Renal Diseases), Taipei, Taiwan; 6 Department of Internal Medicine, National Taiwan University Hospital, Room 1555, B4, Clinical Research Building, 7 Chung-Shan South Road, Taipei, 100 Taiwan

**Keywords:** Type 2 diabetes mellitus, Kidney transplantation, Major adverse cardiovascular events, Major adverse kidney events, Mortality, Glucagon-like peptide 1 receptor agonists

## Abstract

**Background:**

Cardiovascular disease is a leading cause of post-transplant mortality in kidney transplant recipients (KTRs), especially those with diabetes. Although glucagon-like peptide-1 receptor agonists (GLP-1 RAs) have demonstrated cardiovascular and kidney benefits in the general population with type 2 diabetes mellitus (T2DM), evidence regarding their effects in diabetic KTRs is limited.

**Methods:**

This retrospective cohort study utilized data from the Global Collaborative Network in TriNetX, spanning January 1, 2006, to June 1, 2023. Propensity score matching (PSM) with 1:1 ratio was employed to create balanced cohorts. Adult KTRs with T2DM who received GLP-1 RAs within 3 months post-transplant were compared to a matched cohort of KTRs who did not. The primary outcome was all-cause mortality, with secondary outcomes including major adverse cardiovascular events (MACEs) and major adverse kidney events (MAKEs).

**Results:**

A total of 35,488 adult KTRs with T2DM (mean [SD] age, 57.7 [12.2] years; 57.7% men) were identified and 9.8% patients used GLP-1 RAs among 3 months post-transplant. Following PSM, 3564 GLP-1 RAs users were matched with an equal number of nonusers. After a median follow-up of 2.5 years, GLP-1 RAs users had lower risks of mortality (adjusted hazard ratio (aHR), 0.39; 95% CI 0.31–0.50), MACEs (aHR 0.66; 95% CI 0.56–0.79), and MAKEs (aHR 0.66; 95% CI 0.58–0.75). Adverse effects included higher risks of nausea, vomiting and diarrhea, while risks of suicide, hypoglycemia, retinopathy, and pancreatitis were not increased.

**Conclusions:**

In KTRs with T2DM, GLP-1 RAs use was associated with substantial reductions in all-cause mortality, MAKEs, and MACEs compared to nonuse without increasing complications. However, the underutilization of GLP-1 RAs represents a significant opportunity to improve post-transplant outcomes in this high-risk population.

**Graphical abstract:**

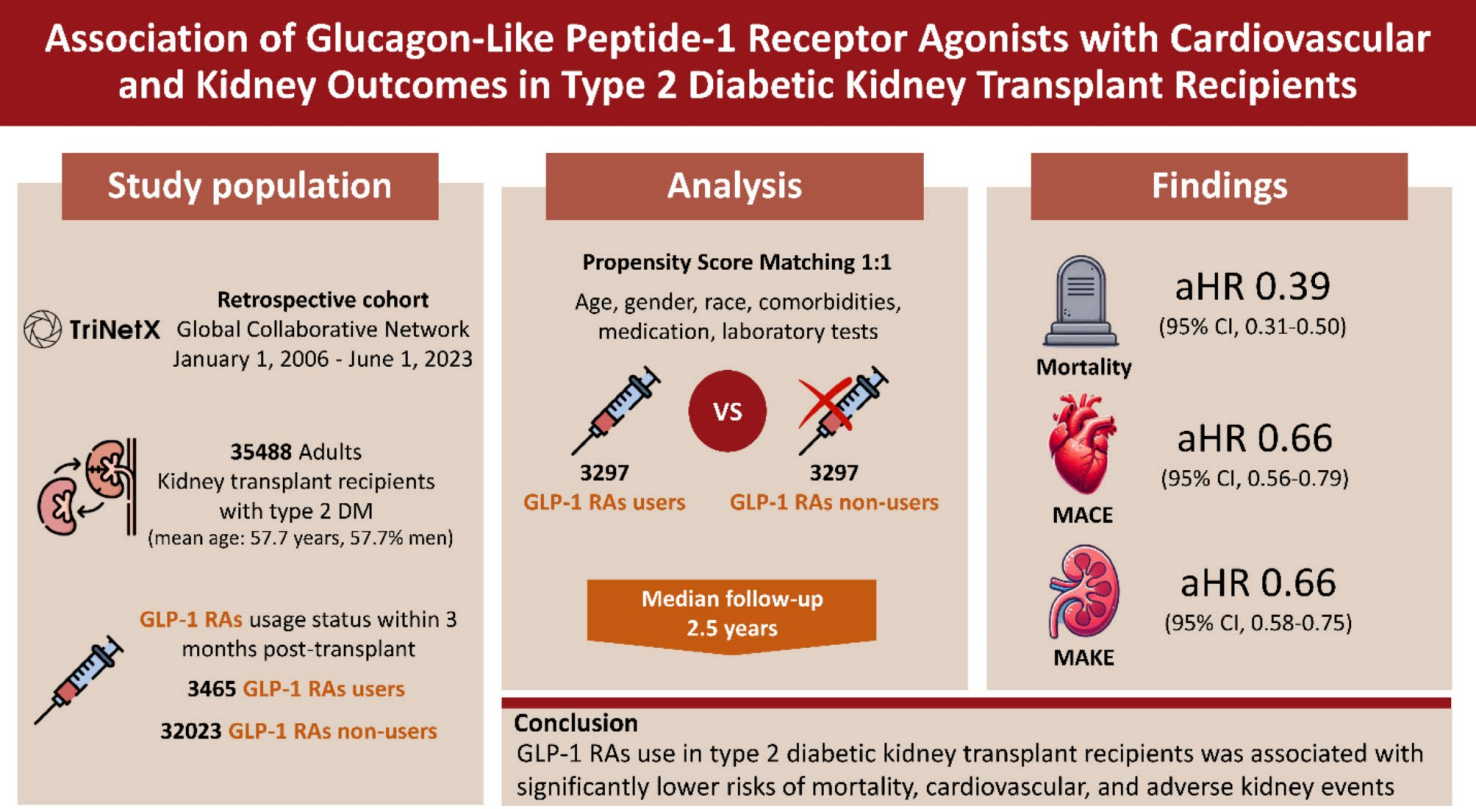

**Supplementary Information:**

The online version contains supplementary material available at 10.1186/s12933-025-02649-0.

## Introduction

Kidney transplantation is recognized as the best treatment option for most patients with end-stage kidney disease (ESKD), as it is associated with reduced risk of mortality and cardiovascular events, and enhanced quality of life compared to maintenance dialysis [1]. Nevertheless, the life expectancy of kidney transplant recipients (KTRs) still falls substantially below that of the general population [2]. Cardiovascular diseases (CVDs) are the leading cause of mortality post-transplant, driven by prevalent traditional risk factors, along with complications related to chronic kidney disease (CKD), such as left ventricular hypertrophy and mineral bone disease, which often persist after the transplant [3, 4]. Diabetes mellitus (DM) is a major risk factor CVDs and is also associated with a higher risk of graft failure [5, 6]. It remains the leading cause of ESKD in adults, with the global prevalence of DM in adult ESKD patients reaching 29.7% in 2015 [7]. Furthermore, retrospective cohort studies identified a 12–38% prevalence of pre-transplant DM [8–10]. In addition to its association with increased mortality, CVDs, and graft failure, pre-transplant DM is also linked to a higher risk of impaired wound healing and infections [11, 12]. Current guidelines recommend screening kidney transplant candidates without known history of DM for abnormal glucose metabolism to enable early detection and management of related complications [13]. Post-transplant diabetes mellitus (PTDM), affecting 5–25% of patients within the first year after transplantation, is another challenge [14]. Both pre-transplant risk factors and the use of immunosuppressants, particularly corticosteroids and calcineurin inhibitors, contribute to the PTDM development [15]. Consequently, optimizing the management in KTRs with DM is of paramount importance to improve long term outcomes.

Glucagon-like peptide-1 receptor agonists (GLP-1 RAs), a class of incretin-based therapies, are promising antidiabetic agents. Over the past decades, several randomized clinical trials (RCTs) have shown that GLP-1 RAs improve cardiovascular outcomes in patients with type 2 DM (T2DM) who have established CVDs or are at risk, as well as in individuals with CKD [16–19]. The American Diabetes Association Standards of Care 2024 guidelines recommends GLP-1 RAs in patients with T2DM who have pre-existing CVDs or are at high risk [20]. Similarly, the KDIGO 2024 CKD guideline recommends GLP-1 RAs for adults with T2DM and CKD who have not achieved individualized glycemic targets [21].

One meta-analysis of observational studies involving 338 KTRs supports the effectiveness of GLP-1 RAs, demonstrating benefits in glycemic control, reductions in proteinuria, and weight loss, without significant interference with tacrolimus blood levels [22]. A retrospective cohort study, by Dotan et al., involving 318 patients, further revealed that treatment with GLP-1 RAs was associated with a reduced risk of major adverse cardiovascular events (MACEs) and all-cause mortality among solid organ transplant recipients, including kidney, lung, liver, and heart transplants [23]. Additionally, Halden et al. demonstrated that GLP-1 infusion improved both insulin secretion and glucagon suppression during hyperglycemia in patients with PTDM [24]. These studies, however, were constrained by small sample sizes and a lack of homogeneity in the cohorts, which included recipients of multiple organ types [15]. The 2024 international PTDM consensus suggested GLP-1 RAs for PTDM patients with obesity and/or established CVDs. Nonetheless, it also acknowledged that these agents remain underutilized in PTDM management due to limitation of transplant-specific evidence [15].

Real-world data offers significant potential for informing and shaping confirmatory trials, enabling the exploration of questions that might otherwise go unanswered. In this study, we utilized the international TriNetX platform to assess whether GLP-1RAs could mitigate long-term adverse outcomes in diabetic KTRs.

## Methods

### Data source

The study utilized data from the TriNetX database, a global collaborative network that integrates de-identified electronic medical records from various healthcare organizations (HCOs), primarily those affiliated with academic medical centers [25–30]. The platform provides detailed patient information, including demographics, diagnoses (coded using the International Classification of Diseases, Tenth Revision, German Modification), procedures (using the International Classification of Diseases, Tenth Revision, Procedure Coding System), medications (based on the Anatomical Therapeutic Chemical Classification or RxNorm codes), along with laboratory results, clinical findings (recorded via local lab coding or Logical Observation Identifiers Names and Codes) and genomic information [31]. We used the Global Collaborative Network, which includes 127 HCOs encompassing over 131 million individuals across 21 countries. The countries included Australia, Belgium, Brazil, Bulgaria, Estonia, France, Georgia, Germany, Ghana, Israel, Italy, Japan, Lithuania, Malaysia, Poland, Singapore, Spain, Taiwan, the United Arab Emirates, the United Kingdom, and the United States. The data was collected over a period spanning from January 1, 2006, to June 1, 2023.

### Ethics statement

The Western Institutional Review Board approved a waiver of informed consent for TriNetX, as the platform solely compiles and presents de-identified data in aggregate form [32]. Additionally, the use of TriNetX for this study was approved by the Institutional Review Board of Chi-Mei Hospital (No: 11210-E01). The study adhered to the principles of the Declaration of Helsinki and followed the STROBE guidelines (Strengthening the Reporting of Observational Studies in Epidemiology) for cohort study reporting [33].

### Study population

The inclusion criteria for the study were as follows: (1) individuals aged over 18 years old, (2) KRTs, and (3) those diagnosed with T2DM either prior to or within 3 months post-transplant. Exclusion criteria included undergoing dialysis or experiencing mortality during 1 to 3 months post-transplant, as delayed graft function rarely persists beyond 1 month [34]. The index date and the time of study entry were defined as the kidney transplant date, identified using ICD-10-CM code Z94.0 and relevant procedure codes. As the intention to treat design, patients were categorized as either GLP-1 RAs users or non-users depending on whether they received these agents within the 3 months following kidney transplant. Due to differences in mechanisms, dual glucose-dependent insulinotropic polypeptide (GIP) and GLP-1 receptor agonists were not included in the definition of GLP-1 RAs users in this study [35–37]. We performed 1:1 propensity score matching (PSM) to generate comparable cohorts.

### Covariates

Clinically relevant covariates, known to influence survival and cardiovascular or kidney outcomes, were carefully selected to ensure balanced comparisons between study groups. Those included various baseline characteristic including demographics (age, gender, race), comorbidities (e.g., liver diseases, chronic lower respiratory diseases, diabetic complications, obesity and neoplasm), medications (e.g., insulin, metformin, angiotensin-converting enzyme inhibitors (ACEIs), angiotensin II receptor blockers (ARBs), tacrolimus, cyclosporine, mycophenolate mofetil (MMF), and corticosteroids), as well as laboratory tests and physical findings (e.g., hemoglobin, glycated hemoglobin (HbA1c) levels, lipid profile, estimated glomerular filtration rate (eGFR) and systolic blood pressure (SBP)). Detailed codes for these covariates are provided in the Supplemental Table 1.

### Prespecified outcomes

The primary outcome is all-cause mortality. Secondary outcomes encompass MACEs, including stroke (either ischemic or hemorrhagic), acute myocardial infarction (AMI), cardiac arrest, cardiogenic shock or death, as well as major adverse kidney events (MAKEs), which is defined as a composite of dialysis dependence, an eGFR less than 15 mL/min/1.73 m², or death. Detailed codes for outcomes are shown in Supplemental Table 2. Outcomes were tracked from 3 months after the index date to a maximum of 5 years. This 3-month window after transplant serves to mitigate reverse causality effects, ensuring that outcomes are more reliably attributed to the use of GLP-1RA. Additionally, it enhances data reliability by avoiding potential reverse etiologies or inconsistencies in immediate post-transplant records. To mitigate protopathic or ascertainment bias, patients with MACEs prior to the study period were excluded, and repeat PSM was performed. Additionally, potential GLP-1RA side effects were assessed for comprehensive safety evaluation.

### Prespecified subgroup, sensitivity and specificity analyses

Subgroup analyses were conducted based on gender, age, body mass index (BMI), HbA1c, DM status (pre-existing or post-transplant), post-transplant eGFR, the presence of hypertension, proteinuria, heart failure, obesity, enrollment time, prior history of GLP-1RAs uses before transplant, and the concurrent use of medications, including ACEIs/ARBs, antidiabetic agents (insulin, metformin, dipeptidyl peptidase-4 inhibitors (DPP-4is), sodium-glucose cotransporter 2 inhibitors (SGLT2is), and immunosuppressants (steroids, cyclosporine and tacrolimus). GLP-1 RA ever users were defined as patients who used GLP-1 RAs within 3 months prior to transplant but discontinued their use afterward. In contrast, new users were those who had not used GLP-1 RAs before transplant and initiated use only after the transplant. Persistent users were identified as individuals who used GLP-1 RAs both before and after the transplant. To further investigate the robustness of our results, we performed sensitivity analyses using the Cox proportional hazards model with alternative covariates and varied cohort exclusion criteria.

Specificity analyses were conducted to evaluate the individual components of the composite outcomes. To enhance the comprehensiveness of our study, we conducted risk analyzed at 1 year and 3 years post-transplant. Additionally, we expanded our comparative analysis of outcomes between two groups of KTRs: those who used GLP-1 RAs in the first three months post-transplant and continued their usage from 3 to 6 months, and those who did not use GLP-1 RAs in the first three months and continued without usage from 3 to 6 months, with outcomes tracked from 6 months post-transplant. Additional specificity analysis was conducted to compare the outcomes of GLP-1 RA users with those of patients receiving other second-line antihyperglycemic treatments, including thiazolidinediones (TZDs), DPP-4is, or sulfonylureas (SUs). Given the established cardiovascular and kidney benefits of SGLT2is for T2DM patients with CVDs, those at high risk, and those with CKD, along with recent cohort studies demonstrating their ability to enhance survival, reduce cardiovascular events, and preserve graft function in kidney KTRs with DM, we conducted additional analyses incorporating SGLT2is [38–42]. Specifically, we compared outcomes between patients who used both GLP-1 RAs and SGLT2is and those who did not receive either medication within the first 3 months post-transplant. To evaluate the impact of GLP-1 RAs on the metabolic profile, we analyzed the serial changes in levels of HbA1c, LDL, body weight, and SBP from different time periods.

### Prespecified positive and negative controls

To evaluate the validity of our approach, we conducted analyses using both positive and negative outcome and exposure controls. We selected nausea, vomiting and diarrhea, widely recognized as adverse effects of GLP-1 RAs, as positive outcome controls [43]. Conversely, sunburn, herniated intervertebral discs, traffic accidents, and pneumonia were chosen as negative outcome controls. For exposure controls, SGLT2is were selected as the positive control, based on evidence from previous cohort studies demonstrating their cardiovascular and kidney benefits in KTRs with DM [44]. On the other hand, topical urea, not expected to influence outcomes, was used as a negative exposure control.

### Landmark analysis

To address immortal time bias, we performed a landmark analysis by refining the cohort selection period, adjusting it from the initial 3 months post-transplant to specific time points within 2, 6, 9, and 12 months post-transplant [45]. This approach ensured that the impact of GLP-1RAs on outcomes remained consistent across different time intervals.

### Statistical analysis

A 1:1 PSM was performed using the aforementioned covariates, utilizing a greedy nearest neighbor algorithm with a 0.1 pooled standard deviation caliper to minimize confounding between the groups. The balance of baseline covariates between the matched populations was evaluated using standardized mean differences, with values below 0.1 indicating a high degree of balance achieved [46]. Baseline characteristics are presented as means with standard deviations (SDs) for continuous variables, and counts with percentages for categorical variables. The Kaplan-Meier method was utilized to estimate overall survival and event-free survival, with the log-rank test assessing statistically significant differences between the two groups. The Cox proportional hazards model was used to calculated adjusted hazard ratios (aHRs) with 95% confidence intervals (CIs) for outcomes associated with the use of GLP-1 RAs. The proportional hazards assumptions were tested using the generalized Schoenfeld approach [47]. Cases with missing outcome data due to loss to follow-up were excluded to prevent bias and inaccuracies arising from incomplete data. To evaluate the impact of unmeasured confounders on the observed relationship between treatment and outcomes, the E-value was used. A large E-value suggests that only very strong unmeasured confounders could negate the observed treatment-outcome association [48]. All statistical analyses were conducted using TriNetX built-in functions and R software (version 4.4.0). A two-sided p-value < 0.05 was considered statistically significant.

## Results

### Baseline characteristics of study population

**Fig. 1:**
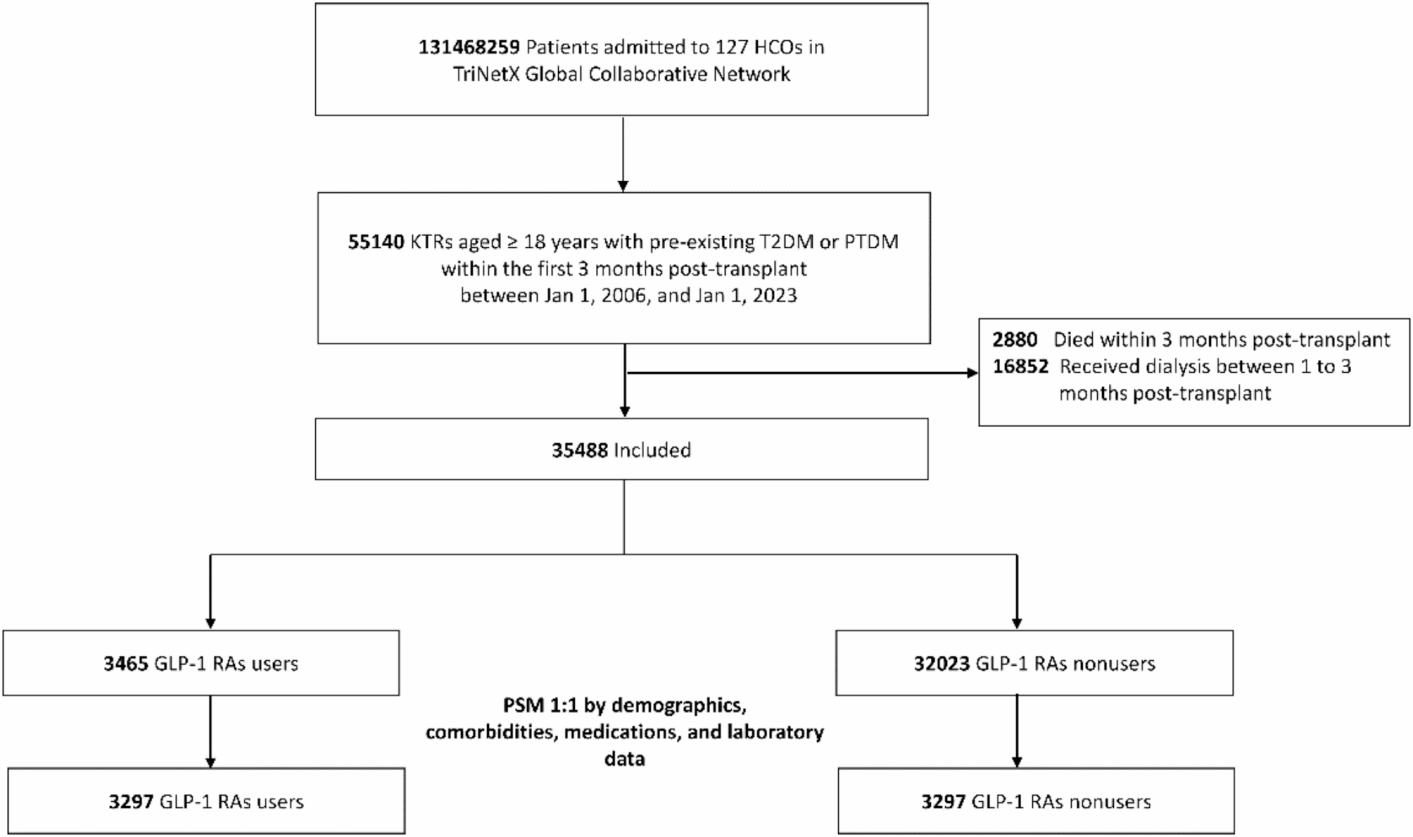
Algorithm for patient selection and enrollment. Abbreviations: GLP-1 RAs, glucagon-like peptide 1 receptor agonists; HCO, healthcare organization; KTR, kidney transplant recipient; PSM, propensity score matching; PTDM, Post-transplant diabetes mellitus; T2DM, type 2 diabetes mellitus

We identified 35,488 adult KTRs with T2DM, with a mean [SD] age of 57.7 [12.2] years and 57.7% being man. Among these, 3,465 (9.8%) received GLP-1 RAs within 3 months post-transplant (Fig. 1). No patients receiving GLP-1 RAs were excluded due to loss of follow-up, while 8 patients (0.2%) who did not receive GLP-1 RAs were excluded for this reason (Supplemental Table 3). Before PSM, significant differences were observed in various covariates. Before PSM, GLP-1 RA users had a smaller proportion of white individuals and were more likely to receive antihypertensive, other antidiabetic agents, and HMG-CoA reductase inhibitors. They exhibited higher HbA1c, higher eGFR, higher BMI, and lower total cholesterol/LDL values. This group also had a higher prevalence of comorbidities, including hypertension, dyslipidemia, heart failure, diabetic complications, overweight and obesity, chronic lower respiratory diseases, cystic kidney disease and neoplasms. Following PSM with 1:1 ratio, 3,297 GLP-1 RA users were matched with an equal number of control patients. After PSM, all standardized differences for the covariates were less than 0.1, indicating that a good balance was achieved (Table 1).

**Table 1:** Baseline characteristics of the study population before and after propensity score matching

	Before matching			After matching		
	GLP-1 RAs user (n = 3465)	Non-users (n = 32,023)	Std diff	GLP-1 RAs users (n = 3297)	Non-users (n = 3297)	Std diff
*Demographics*						
Age, mean ± SD	57.2 ± 11.0	57.7 ± 12.3	0.047	57.2 ± 11.0	57.3 ± 11.7	0.007
Male	1901 (54.9%)	18,572 (58.9%)	0.082	1822 (55.3%)	1812 (55.0%)	0.006
*Race and ethnicity*						
Hispanic or Latino	452 (13.0%)	3236 (10.3%)	0.087	430 (13.0%)	430 (13.0%)	< 0.001
White	1604 (45.6%)	16,485 (49.6%)	0.083	1648 (46.2%)	1615 (45.3%)	0.018
Black or African American	916 (26.4%)	7304 (23.2%)	0.076	859 (26.1%)	825 (25.0%)	0.024
Asian	207 (6.0%)	1901 (6.0%)	0.002	197 (6.0%)	194 (5.9%)	0.004
American Indian or Alaska native	10 (0.3%)	159 (0.5%)	0.082	10 (0.3%)	11 (0.3%)	0.005
Native Hawaiian or other Pacific Islander	10 (0.3%)	284 (0.9%)	< 0.001	10 (0.3%)	10 (0.3%)	< 0.001
*Comorbidities, n (%)*						
Hypertension	2967 (85.6%)	23,117 (73.3%)	0.309	2816 (85.4%)	2806 (85.1%)	0.009
Dyslipidemia	2570 (69.9%)	17,286 (52.2%)	0.379	2478 (69.5%)	2467 (69.2%)	0.007
Overweight and obesity	1405 (40.6%)	6193 (19.6%)	0.468	1301 (39.5%)	1301 (39.5%)	< 0.001
Heart failure	556 (16.1%)	4455 (14.1%)	0.054	528 (16.0%)	569 (17.3%)	0.003
Liver diseases	426 (12.3%)	3765 (11.9%)	0.011	417 (12.6%)	440 (13.3%)	0.021
Chronic lower respiratory diseases	408 (11.8%)	3229 (10.2%)	0.053	382 (11.6%)	396 (12.0%)	0.013
Neoplasms	799 (23.1%)	5995 (19.0%)	0.100	753 (22.8%)	759 (23.0%)	0.011
DM nephropathy	2158 (62.3%)	12,124 (38.5%)	0.491	2026 (61.5%)	2085 (63.2%)	0.037
DM neuropathy	814 (23.5%)	4569 (14.5%)	0.231	775 (23.5%)	795 (24.1%)	0.014
DM ophthalmology	692 (20.0%)	4003 (12.7%)	0.198	647 (19.6%)	661 (20.0%)	0.011
Systemic connective tissue disorders	101 (2.9%)	876 (2.8%)	0.008	96 (2.9%)	100 (3.0%)	0.007
Nephrotic syndrome	54 (1.6%)	451 (1.4%)	0.001	52 (1.6%)	46 (1.4%)	0.015
Cystic kidney disease	213 (6.1%)	1553 (4.9%)	0.054	196 (5.9%)	207 (6.3%)	0.014
Smoking	34 (1.0%)	316 (1.0%)	0.002	32 (1.0%)	28 (0.8%)	0.013
*Medications, n (%)*						
Metformin	600 (17.3%)	1734 (5.5%)	0.378	512 (15.5%)	489 (14.8%)	0.019
Sulfonylureas	511 (14.8%)	2616 (8.3%)	0.203	472 (14.3%)	480 (14.6%)	0.007
Thiazolidinediones	139 (4.0%)	409 (1.3%)	0.170	117 (3.5%)	103 (3.1%)	0.023
DPP4i	562 (16.2%)	1957 (6.2%)	0.322	496 (15.0%)	498 (15.1%)	0.019
SGLT2i	293 (8.4%)	414 (1.3%)	0.336	219 (6.6%)	216 (6.6%)	0.003
Insulin	2616 (75.5%)	17,212 (54.6%)	0.450	2477 (75.1%)	2537 (76.9%)	0.043
ACEI/ARB	1448 (39.4%)	8998 (27.1%)	0.262	1382 (38.8%)	1438 (40.3%)	0.003
Beta-blocker	2382 (68.8%)	18,421 (58.3%)	0.216	2274 (69.0%)	2306 (69.9%)	0.021
CCB	2116 (61.1%)	15,532 (49.3%)	0.239	2018 (61.2%)	2075 (62.9%)	0.036
Diuretics	2016 (58.2%)	15,967 (50.6%)	0.152	1934 (58.7%)	1968 (59.7%)	0.021
HMG-CoA reductase inhibitors	2299 (66.4%)	14,175 (45.0%)	0.441	2157(65.4%)	2184 (66.2%)	0.017
Tacrolimus	2746 (79.3%)	19,931 (63.2%)	0.287	2586 (78.4%)	2632 (79.8%)	0.034
Cyclosporin	124 (3.6%)	1422 (4.5%)	0.047	116 (3.5%)	114 (3.5%)	0.003
MMF	1958 (56.5%)	14,133 (44.8%)	0.236	1858 (56.4%)	1901 (57.7%)	0.026
Azathioprine	102 (2.9%)	860 (2.7%)	0.013	99 (3.0%)	92 (2.8%)	0.013
Corticosteroids	2715 (78.4%)	20,694 (65.6%)	0.287	2586 (78.4%)	2632 (79.8%)	0.034
Sirolimus	93 (2.7%)	787 (2.5%)	0.012	83 (2.5%)	78 (2.4%)	0.010
Everolimus	46 (1.3%)	286 (0.9%)	0.040	43 (1.3%)	39 (1.2%)	0.011
*Laboratory*						
Hb, g/dL	11.9 ± 2.1	11.5 ± 2.2	0.168	11.8 ± 2.1	11.6 ± 2.2	0.099
eGFR, mL/min/1.73m^2^	52.7 ± 21.8	50.7 ± 24.1	0.088	52.4 ± 21.8	51.4 ± 23.1	0.046
HbA1c, %	7.4 ± 1.7	6.8 ± 1.7	0.314	7.3 ± 1.7	7.2. ± 1.7	0.073
Total cholesterol, mg/dL	158.0 ± 43.8	161.0 ± 49.0	0.075	158.0 ± 44.2	156.0 ± 48.8	0.039
LDL cholesterol, mg/dL	79.3 ± 33.9	83.3 ± 37.6	0.110	79.4 ± 34.1	80.3 ± 37.1	0.025
Sodium, mEq/L	139.0 ± 3.1	139.0 ± 3.4	0.004	138.0 ± 3.1	138.0 ± 3.3	0.010
Potassium, mEq/L	4.4 ± 0.5	4.4 ± 0.6	0.013	4.4 ± 0.5	4.4 ± 0.6	0.014
ALT, units/L	24.4 ± 43.0	25.9 ± 30.8	0.042	24.5 ± 43.9	26.5 ± 41.2	0.046
UPCR, mg/g	350.0 ± 1012.0	303 ± 137.0	0.046	353 ± 1036.0	292 ± 730	0.069
BNP, pg/mL	611.0 ± 3237.0	738.0 ± 2309.0	0.045	589.0 ± 3432.0	411.0 ± 935.0	0.062
BMI, kg/m^2^	31.8 ± 5.9	28.9 ± 6.1	0.477	31.7 ± 5.9	31.3 ± 6.2	0.071
SBP, mmHg	134.0 ± 19.2	134.0 ± 20.9	0.007	134.0 ± 19.2	134.0 ± 20.5	0.026

ACEI, angiotensin-converting enzyme inhibitor; ARB, angiotensin receptor blocker; BMI, body mass index; BNP, B-type natriuretic peptide; CCB, calcium channel blocker; DM, diabetes mellitus; DPP-4i, dipeptidyl peptidase-4 inhibitor; eGFR, estimated glomerular filtration rate; GLP-1 RA, glucagon-like peptide 1 receptor agonist; HbA1c, glycated hemoglobin; MMF, Mycophenolate mofetil; SBP, systolic blood pressure; SD, standard deviation; SGLT2i, sodium-glucose cotransporter 2 inhibitor; Std diff, standard difference; UPCR, Urine Protein/Creatinine Ratio.

### Primary outcome and secondary outcomes

**Fig. 2:**
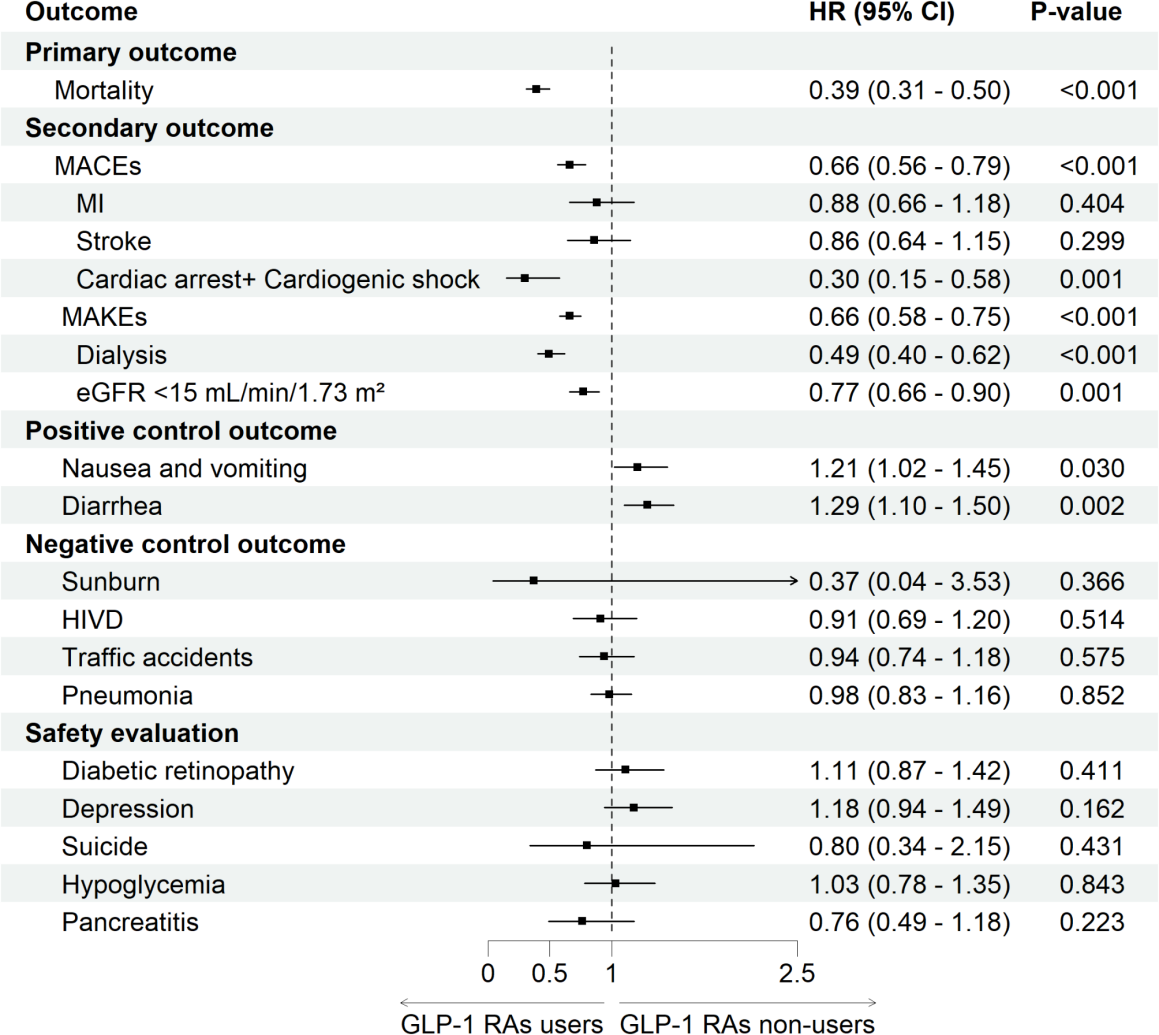
Forest plot of primary and secondary outcomes, positive and negative control outcomes, and safety evaluation. Abbreviation: aHR, adjusted hazard ratio; eGFR; estimated glomerular filtration rate; GLP-1 RA; glucagon-like peptide 1 receptor agonist, HIVD, herniated intervertebral discs; MACE, major adverse cardiac event; MAKE, major adverse kidney events; MI, myocardial infarction

**Fig. 3:**
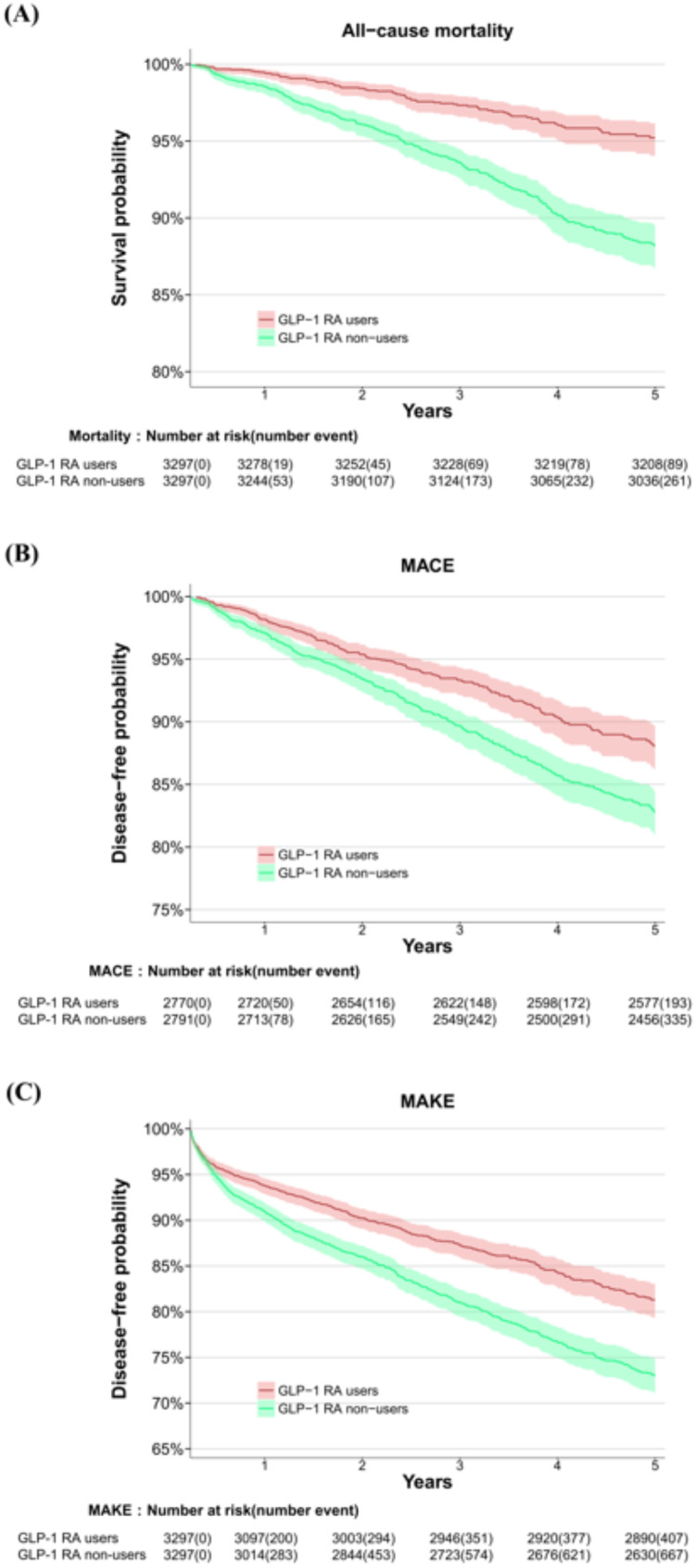
Kaplan–Meier estimates overall and event-free survival according to treatment group among diabetic KTR. **A** all-cause mortality (log-rank *p* < 0.001), **B** MACEs (log-rank *p* < 0.001), **C** MAKEs (log-rank *p* < 0.001). Abbreviations: GLP-1 RA, glucagon-like peptide 1 receptor agonist; KTR, kidney transplant recipients; MACE, major adverse cardiac event; MAKE, major adverse kidney event

After a median duration of follow-up for the whole cohort 2.5 years (interquartile range (IQR), 1.4–3.6 years), 93 patients (2.6%) in the GLP-1 RAs users group and 319 patients (9.0%) in the GLP-1 RAs non-users group experienced all-cause mortality. Use of GLP-1 RAs was associated with a substantially lower risk of all-cause mortality, with an aHR of 0.39 (95% CI 0.31–0.50, *p* < 0.001). Additionally, compared with the non-users, GLP-1 RA users exhibited lower risks of MACEs (7.0% vs. 12.0%; aHR, 0.66; 95% CI 0.56–0.79, *p* < 0.001) and MAKEs (12.3% vs. 20.3%; aHR, 0.66; 95% CI 0.58–0.75, *p* < 0.001) (Figs. 2 and 3, Supplemental Tables 4–6). There were no violations of the proportional hazards assumption (Schoenfeld test *p* = 0.761, 0.816, and 0.697, respectively). The E-values were notably high, at 4.51 for all-cause mortality, 2.38 for MACEs, and 2.00 for MAKEs, indicating that the observed associations are unlikely to be explained away by potential confounding (Supplemental Table 7).

### Subgroup, sensitivity and specificity analyses

**Fig. 4:**
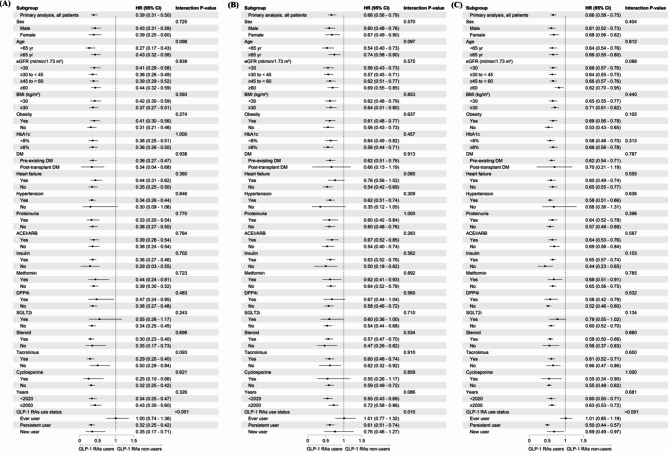
Subgroup analyses. Forest plots of aHRs for GLP-1RAs users versus non-users after kidney transplant, regarding the long-term risks of **A** all-cause mortality, **B** MACEs, **C** MAKEs. Abbreviations: ACEI, angiotensin-converting enzyme inhibitor; ARB, angiotensin receptor blocker; BMI, body mass index; DM, diabetes mellitus; DPP-4i, dipeptidyl peptidase-4 inhibitor; eGFR, estimated glomerular filtration rate; GLP-1 RA; glucagon-like peptide 1 receptor agonist; HR, hazard ratio; MACE, major adverse cardiac event; HbA1c, glycated hemoglobin; MAKE, major adverse kidney event; SGLT2i, sodium-glucose cotransporter 2 inhibitor

Subgroup analyses demonstrated that the benefit of GLP-1 RAs on overall survival, MACEs and MAKEs was broadly consistent across prespecified subgroups. Notably, these advantages persisted irrespective of baseline kidney function, HbA1c levels, BMI, the timing of DM diagnosis, comorbidities and concurrent medication, including immunosuppressants (Fig. 4). Among patients with pre-transplant diabetes, outcomes aligned with the main findings. To account for advancements in medical practice, we performed an additional analysis of patients enrolled before and after 2022. The findings revealed that the benefits of GLP-1 RAs remained consistent across both time periods, unaffected by the chronological changes.

The consistent results from sensitivity analyses using the Cox proportional hazards model with alternative sets of covariates and various exclusion criteria confirmed the robustness of our findings (Supplemental Table 8). Additionally, GLP-1 RAs users consistently demonstrated a statistically significant lower risk of cardiac arrest and cardiogenic shock (aHR = 0.30, *p* = 0.001), dialysis dependence (aHR = 0.49, *p* < 0.001) and eGFR less than 15 mL/min/1.73 m² (aHR = 0.77, *p* < 0.001). While there was a numerical reduction in the incidence of MI (aHR, 0.88; 95% CI 0.66–1.18, *P* = 0.404) and stroke (aHR, 0.86; 95% CI 0.64–1.15, *p* = 0.299) among GLP-1 RAs users, these differences did not reach statistical significance (Fig. 2). Outcome analysis conducted at 1 year or 3 years post-transplant showed consistent results. Comparative analysis of outcomes between those who used GLP-1 RAs in the first 3 months post-transplant and continued from 3 to 6 months, versus those who did not use GLP-1 RAs during either period, yielded similar results (Supplemental Table 9). Similarly, outcomes were consistent when comparing patients who used both GLP-1 RAs and SGLT2 is with those who did not receive either medication within the first 3 months post-transplant (Supplemental Table 10). Additionally, when comparing the outcomes of GLP-1 RAs users with those receiving DPP-4is, TZDs, or SUs, the results remained consistent (Supplemental Table 11). Serial analyses of the metabolic profile showed consistently high HbA1c levels and body weight loss among GLP-1 RAs users. However, LDL levels and SBP did not made significant different (Supplemental Table 12). However, landmark analysis, adjusting the cohort selection period to 2, 6, 9, and 12 months post-transplant, demonstrated consistent beneficial effects of GLP-1 RAs on all-cause mortality, MACEs, and MAKEs (Supplemental Table 13).

### Positive and negative controls outcome

As expected, the positive results confirmed that the use of GLP-1 RAs was associated with an increased incidence of nausea/vomiting (aHR, 1.21; 95% CI 1.02–1.45, *p* = 0.030 and diarrhea (aHR, 1.29; 95% CI 1.10–1.50, *p* = 0.002). Additionally, no association was observed between the use of GLP-1 RAs and the incidence of sunburn, herniated intervertebral discs, traffic accidents, or pneumonia. Further safety evaluations revealed that the risks of suicide, depression, hypoglycemia, retinopathy, and pancreatitis were not increased with the use of GLP-1 RAs (Fig. 2).

### Positive and negative exposure controls

We observed that SGLT2is users experienced substantial reductions in all-cause mortality (aHR, 0.44; 95% CI 0.33–0.60, *p* < 0.001), MACEs (aHR, 0.68; 95% CI 0.54–0.85, *p* < 0.001) and MAKEs (aHR, 0.62; 95% CI 0.53–0.73, *p* < 0.001) in T2DM after kidney transplants. In contrast, topical urea did not show any significant effects (Supplemental Table 14).

## Discussion

In this study, we observed only a small proportion (9.8%) of adult KTRs with T2DM being treated with GLP-1 RAs among 3 months after transplant. Our analysis demonstrated that the use of GLP-1 RAs was associated with a significantly decreased risk of all-cause mortality, MACEs, and MAKEs over a median follow-up period of 2.5 years. These benefits were consistently observed across diverse subgroups, regardless of obesity, baseline kidney function, or glucose control. Notably, persistent or new users of GLP-1 RAs exhibited better survival and kidney outcomes compared to ever users, with persistent users also showing superior MACEs and MAKEs outcomes. While gastrointestinal side effects like nausea and vomiting were more common, there was no increased risk of hypoglycemia, suicide, or pancreatitis, underscoring the safety of GLP-1 RAs in this population. These results confirm the efficacy and safety of GLP-1 RAs for managing T2DM in KTRs.

Our observations about the efficacy of GLP-1 RAs in enhancing survival and reducing cardiovascular events are concordance with evidence from several cardiovascular outcome trials (CVOTs) conducted in general population with T2DM, as well as a recent cohort study focused on solid organ transplant recipients [16–19, 23]. Moreover, the observed high incidence of MACEs, which reached 12.0% in the non-users during follow-up, highlights the elevated cardiovascular risk among KTRs with T2DM, underscoring the importance of incorporating GLP-1 RAs into the DM care for these specific population.

The cardioprotective effects of GLP-1 RAs are thought to arise from their pleiotropic effects [49]. This is supported by the widespread expression of GLP-1 receptors outside the pancreas, such as in the brain, lungs, heart, kidneys, liver, nervous system, endothelial cells, and immune cells [50]. Preclinical studies indicate that GLP-1 RAs can attenuate atherosclerosis by reducing vascular inflammation, lowering oxidative stress, and inhibiting the proliferation and activation of vascular smooth muscle cells [51, 52]. Additionally, these agents have been shown to decrease epicardial fat deposits, reduce cardiomyocyte apoptosis, optimize cardiac energetics, and enhance myocardial glucose oxidation [53, 54]. The consistently high HbA1c levels observed among GLP-1 RAs users in our study further reinforce their pleiotropic actions beyond glucose lowering [55].

Secondary endpoints in CVOTs with GLP-1 RAs have also suggested potential kidney benefits, primarily through reductions in albuminuria, with some trials reporting a mitigation of eGFR decline [16–19, 56, 57]. The recently published FLOW trial, which specifically targeted T2DM patients with CKD and used kidney-specific primary endpoints, has solidified the efficacy of GLP-1 RAs in slowing CKD progression [58]. Regarding their use in KTRs with T2DM, data remain sparse. A meta-analysis of nine observational studies, involving 338 KTRs with a median follow-up time of 12 months, found that treatment with GLP-1 RAs was associated with a decrease in proteinuria but did not significantly alter eGFR levels [22]. Notably, one study included in the meta-analysis, with a follow-up period of at least 2 years, showed that GLP-1 RAs were associated with a lower risk of MAKEs, and protocol biopsies results indicated that GLP-1 RAs may promote tubular cell regeneration in the kidney graft [59]. The significant reduction in the risk of MAKEs observed in our study could be attributed to the relatively longer follow-up period and the larger number of participants. The gastrointestinal effects associated with GLP-1 RAs, such as delayed gastric emptying, nausea, and vomiting, raise concerns about potential interference with the therapeutic level immunosuppressants, possibly leading to graft failure. Although we were unable to assess blood drugs levels in the TriNetX database, the consistent reduction in MAKEs observed in steroid, tacrolimus and cyclosporine subgroups provides indirect reassurance of the safety of GLP-1 RAs in KRTs and regardless of the specific immunosuppressive regimen. These findings align with previous studies, which have shown that GLP-1 RAs do not significantly affect tacrolimus trough levels or lead to acute rejection [60–62].

The precise mechanism of kidney-protective effects of GLP-1 RAs still remains elusive, but likely include direct kidney actions such as inducing natriuresis through NHE3 inhibition in the proximal tubules, suppressing the intrarenal renin-angiotensin system, ameliorating kidney ischemia/hypoxia [63, 64]. These direct effects are further supported by human studies demonstrating the wide expression of GLP-1 receptors in the proximal tubules, juxtaglomerular cells, hilar and intralobular arteries, and preglomerular vascular smooth muscle cells [49]. Additionally, GLP-1 RAs have shown potential in mitigating inflammation in diabetic kidney disease by inhibiting angiotensin II signaling, downregulating the receptor for advanced glycation end products, attenuating myelopoiesis, and promoting M2 macrophage polarization in mouse models [65, 66].

To our knowledge, this study represents the largest cohort to date investigating the association between GLP-1 RAs and cardiovascular and kidney outcomes in KTRs with T2DM. Additionally, the strengths of our study include the long follow-up period and the comprehensive validation of results through various methods, such as sensitivity and specificity analyses. However, this study has several limitations. First, as with other studies using electronic health databases, the analysis of the TriNetX database relies heavily on diagnosis codes, which may introduce both misclassification bias due to coding errors and ascertainment bias from the underrepresentation of mild cases. Additionally, we were unable to evaluate the exact reasons for drug prescriptions, medication switches, or adherence. Nevertheless, the study employed an intention-to-treat design, and our analysis was structured to ensure consistent results, thereby reducing the potential for guarantee-time or immortal time bias [67]. The specificity tests revealed no difference between patients with GLP-1RAs users in our negative control analysis, aiding in the removal of selection bias that can be caused by existing knowledge of an individual’s assignment. Second, although we employed PSM to balance covariates between treated and control groups, residual confounding could not be completely avoided. However, the high E-values from our analysis suggest that the likelihood of residual confounders explaining the observed associations is very low, thereby strengthening the potential association in our findings. Additionally, we employed various medications and biochemical data as proxies for disease severity, helping to mitigate the inherent limitations of using electronic health records. Third, the built-in statistical platform in TriNetX restricted the possibility of performing more refined analyses, which cannot be entirely eliminated despite the implementation of rigorous methodologies and variable validation strategies. Forth, the aggregate nature of the data limited our capacity to explore the severity of gastrointestinal adverse effects and their association with drug discontinuation. Fifth, previous CVOTs have suggested that the beneficial effects of GLP-1 RAs are primarily associated with long-acting agents such as liraglutide, semaglutide, and dulaglutide, rather than short-acting agents like exenatide and lixisenatide [17, 54, 56, 57, 68]. However, the lack of detailed data on specific GLP-1 RAs types limited the scope of subgroup analyses. Similarly, the limited number of records for specific dose of GLP-1 RAs precluded an assessment of dose-response effects. Sixth, we were unable to evaluate changes in insulin resistance following the use of GLP-1 RAs due to the limited availability of data on insulin resistance indices in the TriNetX database. Seventh, the TriNetX dataset lacks detailed information on the causes of death, which limits our ability to evaluate kidney-specific or cardiovascular mortality. Finally, before PSM, GLP-1 RAs users exhibited a higher prevalence of comorbidities and higher prescription rates for several medications. This imbalance could lead to potential misestimation of GLP-1 RA effects when generalized to all KTRs with T2DM.

## Conclusions

This study represents the largest cohort to date showing that GLP-1 RAs use in KTRs with T2DM is associated with reduced risks of mortality, as well as adverse cardiovascular and kidney outcomes, compared to nonuse. The underutilization of GLP-1 RAs presents an opportunity to improve outcomes in this high-risk population without increased complications. Further RTCs are warranted to validate these findings and identify specific subgroups of KTRs who would benefit most from GLP-1 RA therapy.

## Electronic supplementary material

Below is the link to the electronic supplementary material.

Supplementary Material 1

## Data Availability

No datasets were generated or analysed during the current study.

## Abbreviations

ACEI: Angiotensin-converting enzyme inhibitors
aHR: Adjusted hazard ratio
AMI: Acute myocardial infarction
ARB: Angiotensin II receptor blockers
BMI: Body mass index
CVD: Cardiovascular diseases
CVOT: Cardiovascular outcome trial
CI: Confidence interval
CKD: Chronic kidney disease
DM: Diabetes mellitus
DPP-4i: Dipeptidyl peptidase-4 inhibitor
eGFR: Estimated glomerular filtration rate
ESKD: End-stage kidney disease
GLP-1 RA: Glucagon-like peptide-1 receptor agonist
HbA1c: Glycated hemoglobin
HCOs: Healthcare organizations
IQR: Interquartile range
KTR: Kidney transplant recipient
MACE: Major adverse cardiovascular events
MAKE: Major kidney event
PSM: Propensity score matching
PTDM: Post-transplant diabetes mellitus
RCTs: Randomized clinical trials
SBP: Systolic blood pressure
SD: Standard deviation
SGLT2i: Sodium-glucose cotransporter 2 inhibitor
SU: Sulfonylureas
T2DM: Type 2 diabetes mellitus
TZD: Thiazolidinediones
