# Supplementary material for: Association of glucagon-like peptide-1 receptor agonists with cardiovascular and kidney outcomes in type 2 diabetic kidney transplant recipients
Source: Cardiovasc Diabetol. 2025 Feb 21;24:87. doi: 10.1186/s12933-025-02649-0 (PMC11846168; doi:10.1186/s12933-025-02649-0)
Supplement: Supplementary file 1 — Supplementary Material 1 [file 12933_2025_2649_MOESM1_ESM.docx]

**Supplementary Online Content**

1. Supplementary table

**Supplemental Table 1.** Demographic, diagnostic, medication and laboratory codes used in the definition of covariates

**Supplemental Table 2.** Outcome definition

**Supplemental Table 3.** Numbers and baseline characteristics of GLP-1 RAs non-user with and without follow-up

**Supplemental Table 4.** Risk of all-cause mortality in KRTs with T2DM: comparison between GLP-1 RAs users and non-users after propensity score matching

**Supplemental Table 5.** Risk of MACEs in KRTs with T2DM: comparison between GLP-1 RAs users and non-users after propensity score matching

**Supplemental Table 6.** Risk of MAKEs in KRTs with T2DM: comparison between GLP-1 RAs users and non-users after propensity score matching

**Supplemental Table 7.** Incidence rate ratios and E-values of outcomes of interest among the GLP-1 RAs users compared to the control group after propensity score matching

**Supplemental Table 8.** Sensitivity analysis for all-cause mortality, MACEs and MAKEs between GLP-1 RAs user and GLP-1 RAs non-user

**Supplemental Table 9.** Specificity analysis for all-cause mortality, MACEs and MAKEs between GLP-1 RAs user and GLP-1 RAs non-user

**Supplemental Table 10.** Specificity analysis for all-cause mortality, MACEs and MAKEs between GLP-1 RAs user and patients using DPP-4is, TZDs or SUs

**Supplemental Table 11.** Specificity analysis for all-cause mortality, MACEs and MAKEs between patients receiving both GLP-1 RAs and SGLT2is versus those who did not receive either medication

**Supplemental Table 12.** Comparative HbA1c, body weight, LDL, and SBP between GLP

1 RAs user and GLP-1 RAs non-user

**Supplemental Table 13.** Landmark analysis

**Supplemental Table 14.** Positive and negative exposure controls

1. Supplementary figure

**Supplemental Figure 1.** Graphic abstract

1. STROBE statement checklist
2. Supplementary table

**Supplemental Table 1.** Demographic, diagnostic, medication and laboratory codes used in the definition of covariates

| **Category** | **Code** | **Description** |
| --- | --- | --- |
| Demographics | AI | Age at index |
| Demographics | M | Male |
| Demographics | 2106-3 | White |
| Demographics | 2054-5 | Black or African American |
| Demographics | 2106-3 | White |
| Demographics | 2028-9 | Asian |
| Demographics | 2135-2 | Hispanic or Latino |
| Demographics | 1002-5 | American Indian or Alaska Native |
| Demographics | 2076-8 | Native Hawaiian or Other Pacific Islander |
| Diagnosis | I10 | Essential hypertension |
| Diagnosis | I50 | Heart failure |
| Diagnosis | E11.2 | Type 2 diabetes mellitus with kidney complications |
| Diagnosis | E11.3 | Type 2 diabetes mellitus with ophthalmic complications |
| Diagnosis | E11.4 | Type 2 diabetes mellitus with neurological complications |
| Diagnosis | K70-K77 | Diseases of liver |
| Diagnosis | J40-J4A | Chronic lower respiratory diseases |
| Diagnosis | M30-M36 | Systemic connective tissue disorders |
| Diagnosis | C00-D49 | Neoplasms |
| Diagnosis | E78 | Disorders of lipoprotein metabolism and other lipidemias |
| Diagnosis | E66 | Overweight and obesity |
| Diagnosis | N04 | Nephrotic syndrome |
| Diagnosis | Q61 | Cystic kidney disease |
| Diagnosis | Z72.0 | Tobacco use |
| Medication | A10A | Insulins and analogues |
| Medication | A10BA | Biguanides |
| Medication | A10BB | Sulfonylureas |
| Medication | A10BG | Thiazolidinediones |
| Medication | A10BH | Dipeptidyl peptidase 4 (dpp-4) inhibitors |
| Medication | A10BK | Sodium-glucose co-transporter 2 (sglt2) inhibitors |
| Medication | C10AA | Hmg coa reductase inhibitors |
| Medication | C03 | Diuretics |
| Medication | C07 | Beta blocking agents |
| Medication | C08 | Calcium channel blockers |
| Medication | C09 | Agents acting on the renin-angiotensin system |
| Medication | H02 | Corticosteroids for systemic use |
| Medication | 1256 | Azathioprine |
| Medication | 3008 | Cyclosporine |
| Medication | 35302 | Sirolimus |
| Medication | 42316 | Tacrolimus |
| Medication | 68149 | Mycophenolate mofetil |
| Medication | 141704 | Everolimus |
| Laboratory | 8001 | Glomerular filtration rate/1.73 sq M.predicted [Volume Rate/Area] in Serum, Plasma or Blood by Creatinine-based formula (MDRD) |
| Laboratory | 9000 | Cholesterol [Mass/volume] in Serum or Plasma |
| Laboratory | 9002 | Cholesterol in LDL [Mass/volume] in Serum or Plasma |
| Laboratory | 9003 | Natriuretic peptide B [Mass/volume] in Serum, Plasma or Blood |
| Laboratory | 9014 | Hemoglobin [Mass/volume] in Blood |
| Laboratory | 9028 | Potassium [Moles/volume] in Serum, Plasma or Blood |
| Laboratory | 9029 | Sodium [Moles/volume] in Serum, Plasma or Blood |
| Laboratory | 9037 | Hemoglobin A1c/Hemoglobin.total in Blood |
| Laboratory | 9044 | Alanine aminotransferase [Enzymatic activity/volume] in Serum, Plasma or Blood |
| Laboratory | 9045 | Albumin [Mass/volume] in Serum, Plasma or Blood |
| Laboratory | 2890-2 | Protein/Creatinine [Mass Ratio] in Urine |
| Clinical | 9083 | BMI |
| Clinical | 9085 | Blood Pressure, Systolic |

**Supplemental Table 2. Outcome definition**

| Mortality | | | | |
| --- | --- | --- | --- | --- |
|  | **Outcome definition** | | | |
|  | | Diagnosis | UMLS:ICD10CM:R99 | Ill-defined and unknown cause of mortality |
|  | | Demographics | Deceased | Deceased |
|  | |  |  |  |
| MACE | | | | |
|  | **Outcome definition** | | | |
|  | | Demographics | Deceased | Deceased |
|  | | Diagnosis | UMLS:ICD10CM:R99 | Ill-defined and unknown cause of mortality |
|  | | Diagnosis | UMLS:ICD10CM:I21 | Acute myocardial infarction |
|  | | Diagnosis | UMLS:ICD10CM:I46 | Cardiac arrest |
|  | | Diagnosis | UMLS:ICD10CM:I61 | Nontraumatic intracerebral hemorrhage |
|  | | Diagnosis | UMLS:ICD10CM:I63 | Cerebral infarction |
|  | |  |  |  |
| MAKE | | | | |
|  | **Outcome definition** | | | |
|  | | Demographics | Deceased | Deceased |
|  | | Diagnosis | UMLS:ICD10CM:R99 | Ill-defined and unknown cause of mortality |
|  | | Procedure | UMLS:CPT:90937 | Hemodialysis procedure requiring repeated evaluation(s) with or without substantial revision of dialysis prescription |
|  | | Procedure | UMLS:CPT:90947 | Dialysis procedure other than hemodialysis (eg, peritoneal dialysis, hemofiltration, or other continuous renal replacement therapies) requiring repeated evaluations by a physician or other qualified health care professional, with or without substantial revision of dialysis prescription |
|  | | Procedure | UMLS:CPT:90945 | Dialysis procedure other than hemodialysis (eg, peritoneal dialysis, hemofiltration, or other continuous renal replacement therapies), with single evaluation by a physician or other qualified health care professional |
|  | | Procedure | UMLS:CPT:1012752 | Hemodialysis Procedures |
|  | | Procedure | UMLS:ICD9CM:39.95 | Hemodialysis |
|  | | Procedure | UMLS:CPT:90935 | Hemodialysis procedure with single evaluation by a physician or other qualified health care professional |
|  | | Procedure | UMLS:SNOMED:302497006 | Hemodialysis |
|  | | Procedure | UMLS:CPT:1012740 | Dialysis Services and Procedures |
|  | | Diagnosis | UMLS:ICD10CM:Z99.2 | Dependence on renal dialysis |
|  | | Laboratory | TNX:8001 | Glomerular filtration rate/1.73 sq M.predicted [Volume Rate/Area] in Serum, Plasma or Blood by Creatinine-based formula (MDRD) (between 0 and 15.00 mL/min/1.73m^2^ (most recent occurrence)) |
|  | |  |  |  |
| Nausea and vomiting | | | | |
|  | **Outcome definition** | | | |
|  | | Diagnosis | UMLS:ICD10CM:R11 | Nausea and vomiting |

| Diarrhea | | | | |
| --- | --- | --- | --- | --- |
|  | **Outcome definition** | | | |
|  | | Diagnosis | UMLS:ICD10CM:R19.7 | Diarrhea, unspecified |

| Sunburn | | | | |
| --- | --- | --- | --- | --- |
|  | **Outcome definition** | | | |
|  | | Diagnosis | UMLS:ICD10CM:L55 | Sunburn |

| Herniated disc | | | | | | | | | |
| --- | --- | --- | --- | --- | --- | --- | --- | --- | --- |
|  | **Outcome definition** | | | | | | | | |
|  | | | | Diagnosis | | | UMLS:ICD10CM:M51 | Thoracic, thoracolumbar, and lumbosacral intervertebral disc disorders | |
| Pneumonia | | | | | | | | | |
|  | **Outcome definition** | | | | | | | | |
|  | | | Diagnosis | | | UMLS:ICD10CM:J18 | | | Pneumonia, unspecified organism |
|  | | Diagnosis | | | UMLS:ICD10CM:J18.9 | | | | Pneumonia, unspecified organism |
|  | | Diagnosis | | | UMLS:ICD10CM:J12.82 | | | | Pneumonia due to coronavirus disease 2019 |
|  | | Diagnosis | | | UMLS:ICD10CM:J13 | | | | Pneumonia due to Streptococcus pneumoniae |
|  | | Diagnosis | | | UMLS: ICD10CM:J14 | | | | Pneumonia due to Hemophilus influenzae |
|  | | Diagnosis | | | UMLS:ICD10CM:J15.0 | | | | Pneumonia due to Klebsiella pneumoniae |
|  | | Diagnosis | | | UMLS:ICD10CM:J15.1 | | | | Pneumonia due to Pseudomonas |
|  | | Diagnosis | | | UMLS:ICD10CM:J15.2 | | | | Pneumonia due to staphylococcus |
|  | | Diagnosis | | | UMLS:ICD10CM:J15.21 | | | | Pneumonia due to staphylococcus aureus |
|  | | Diagnosis | | | UMLS:ICD10CM:J15.211 | | | | Pneumonia due to Methicillin susceptible Staphylococcus aureus |
|  | | Diagnosis | | | UMLS:ICD10CM:J15.212 | | | | Pneumonia due to Methicillin resistant Staphylococcus aureus |
|  | | Diagnosis | | | UMLS:ICD10CM:J15.4 | | | | Pneumonia due to other streptococci |
|  | | Diagnosis | | | UMLS:ICD10CM:J15.6 | | | | Pneumonia due to other Gram-negative bacteria |
|  | | Diagnosis | | | UMLS:ICD10CM:J15.8 | | | | Pneumonia due to other specified bacteria |
|  | | Diagnosis | | | UMLS:ICD10CM:J16 | | | | Pneumonia due to other infectious organisms, not elsewhere classified |

| Diabetic retinopathy | | | | |
| --- | --- | --- | --- | --- |
|  | **Outcome definition** | | | |
|  | | Diagnosis | UMLS:ICD10CM:E11.3 | Type 2 diabetes mellitus with ophthalmic complications |
|  | | Diagnosis | UMLS:ICD10CM:E11.31 | Type 2 diabetes mellitus with unspecified diabetic retinopathy |
|  | | Diagnosis | UMLS:ICD10CM:E11.311 | Type 2 diabetes mellitus with unspecified diabetic retinopathy with macular edema |
|  | | Diagnosis | UMLS:ICD10CM:E11.319 | Type 2 diabetes mellitus with unspecified diabetic retinopathy without macular edema |
|  | | Diagnosis | UMLS:ICD10CM:E11.32 | Type 2 diabetes mellitus with mild nonproliferative diabetic retinopathy |
|  | | Diagnosis | UMLS:ICD10CM:E11.321 | Type 2 diabetes mellitus with mild nonproliferative diabetic retinopathy with macular edema |
|  | | Diagnosis | UMLS:ICD10CM:E11.329 | Type 2 diabetes mellitus with mild nonproliferative diabetic retinopathy without macular edema |
|  | | Diagnosis | UMLS:ICD10CM:E11.3293 | Type 2 diabetes mellitus with mild nonproliferative diabetic retinopathy without macular edema, bilateral |
|  | | Diagnosis | UMLS:ICD10CM:E11.33 | Type 2 diabetes mellitus with moderate nonproliferative diabetic retinopathy |
|  | | Diagnosis | UMLS:ICD10CM:E11.331 | Type 2 diabetes mellitus with moderate nonproliferative diabetic retinopathy with macular edema |
|  | | Diagnosis | UMLS:ICD10CM:E11.339 | Type 2 diabetes mellitus with moderate nonproliferative diabetic retinopathy without macular edema |
|  | | Diagnosis | UMLS:ICD10CM:E11.34 | Type 2 diabetes mellitus with moderate nonproliferative diabetic retinopathy without macular edema |
|  | | Diagnosis | UMLS:ICD10CM:E11.349 | Type 2 diabetes mellitus with severe nonproliferative diabetic retinopathy |
|  | | Diagnosis | UMLS:ICD10CM:E11.35 | Type 2 diabetes mellitus with proliferative diabetic retinopathy |
|  | | Diagnosis | UMLS:ICD10CM:E11.351 | Type 2 diabetes mellitus with proliferative diabetic retinopathy with macular edema |
|  | | Diagnosis | UMLS:ICD10CM:E11.359 | Type 2 diabetes mellitus with proliferative diabetic retinopathy without macular edema |
|  | | Diagnosis | UMLS:ICD10CM:E11.3593 | Type 2 diabetes mellitus with proliferative diabetic retinopathy without macular edema, bilateral |
|  | | Diagnosis | UMLS:ICD10CM:E11.3599 | Type 2 diabetes mellitus with proliferative diabetic retinopathy without macular edema, unspecified eye |

| Depression | | | | | | |
| --- | --- | --- | --- | --- | --- | --- |
|  | **Outcome definition** | | | | | |
|  | | | Diagnosis | | UMLS:ICD10CM:F32 | Depression episode |
|  | | Diagnosis | | UMLS:ICD10CM:F32.A | | Depression episode, unspecified |

| Suicide | | | | | | |
| --- | --- | --- | --- | --- | --- | --- |
|  | **Outcome definition** | | | | | |
|  | | | Diagnosis | | UMLS:ICD10CM:T14.91 | Suicide attempt |
|  | | Diagnosis | | UMLS:ICD10CM:T14.91XA | | Suicide attempt. Initial encounter |
|  | | Diagnosis | | UMLS:ICD10CM:T14.91XD | | Suicide attempt. subsequent encounter |

| Hypoglycemia | | | | |
| --- | --- | --- | --- | --- |
|  | **Outcome definition** | | | |
|  | | Diagnosis | UMLS:ICD10C:E16.2 | Hypoglycemia, unspecified |

| Pancreatitis | | | | |
| --- | --- | --- | --- | --- |
|  | **Outcome definition** | | | |
|  | | Pancreatitis | UMLS:ICD10C:K85 | Acute pancreatitis |

**Supplemental Table 3.** Numbers and baseline characteristics of GLP-1 RAs non-user with and without follow-up

|  | **GLP-1 RAs non-user with follow-up** | **GLP-1 RAs non-user without follow-up** |
| --- | --- | --- |
| **Number of patients** | 32023 | 8 |
| **Demographics** | | |
| Age, mean ± SD | 57.7 ± 12.3 | 64.0 ± 13.9 |
| Male | 18572 (58.9%) | 5 (62.5%) |
| **Race and ethnicity** |  |  |
| Hispanic or Latino | 3236 (10.3%) | 4 (50.0%) |
| White | 16485 (49.6%) | 1 (12.5%) |
| Black or African American | 7304 (23.2%) | 2 (25%) |
| Asian | 1901 (6.0%) | 1 (12.5%) |
| American Indian or Alaska Native | 159 (0.5%) | 0 (0%) |
| Native Hawaiian or Other Pacific Islander | 284 (0.9%) | 0 (0%) |
| **Comorbidities** |  |  |
| Hypertension | 23117 (73.3%) | 6 (75.0%) |
| Dyslipidemia | 17286 (52.2%) | 3 (37.5%) |
| Overweight and obesity | 6193 (19.6%) | 3 (37.5%) |
| Heart failure | 4455 (14.1%) | 5 (62.5%) |
| Liver diseases | 3765 (11.9%) | 3 (37.5%) |
| Chronic lower respiratory diseases | 3229 (10.2%) | 3 (37.5%) |
| Neoplasms | 5995 (19.0%) | 4 (50%) |
| Diabetic nephropathy | 12124 (38.5%) | 3 (37.5%) |
| Diabetic neuropathy | 4569 (14.5%) | 0 (0%) |
| Diabetic ophthalmology | 4003 (12.7%) | 0 (0%) |
| Systemic connective tissue disorders | 876 (2.8%) | 1 (12.5%) |
| Nephrotic syndrome | 451 (1.4%) | 0 (0%) |
| Cystic kidney disease | 1553 (4.9%) | 0 (0%) |
| Smoking | 316 (1.0%) | 0 (0%) |

**Abbreviation:** GLP-1 RAs, glucagon-like peptide 1 receptor agonists; SD, standard deviation

# **Supplemental Table 4.** Risk of all-cause mortality in KRTs with T2DM: Comparison between GLP-1 RAs users and non-users after propensity score matching

# This table presents the outcome analysis of kidney transplant recipients with T2DM, comparing those who received GLP-1 RAs (Cohort 1) with those who did not receive GLP-1 RAs (Cohort 2) after propensity score matching.

| **Mortality** | | | | | | | | | | | | |
| --- | --- | --- | --- | --- | --- | --- | --- | --- | --- | --- | --- | --- |
|  | | **Risk analysis** | | | | | | | | | | |
|  |  | | | Cohort | | | Patients in cohort | Patients with outcome | Risk | | | |
|  | | |  | 1 | | KTRDM+GLP-1 RAs | 3,297 | 89 | 0.027 | | | |
|  | | |  | 2 | | KTRDM-GLP-1 RAs | 3,297 | 261 | 0.079 | | | |
|  | | | | | | | | | | | | |
|  | | |  |  | | |  | 95% CI | z | p |  |  |
|  | | |  | **Risk Difference** | | | -0.052 | (-0.063, -0.041) | -9.448 | 0.000 |  |  |
|  | | |  | **Risk Ratio** | | | 0.341 | (0.269, 0.432) | N/A | N/A |  |  |
|  | | |  | **Odds Ratio** | | | 0.323 | (0.252, 0.413) | N/A | N/A |  |  |
|  | | | | | | | | | | | | |
|  | |  | | | 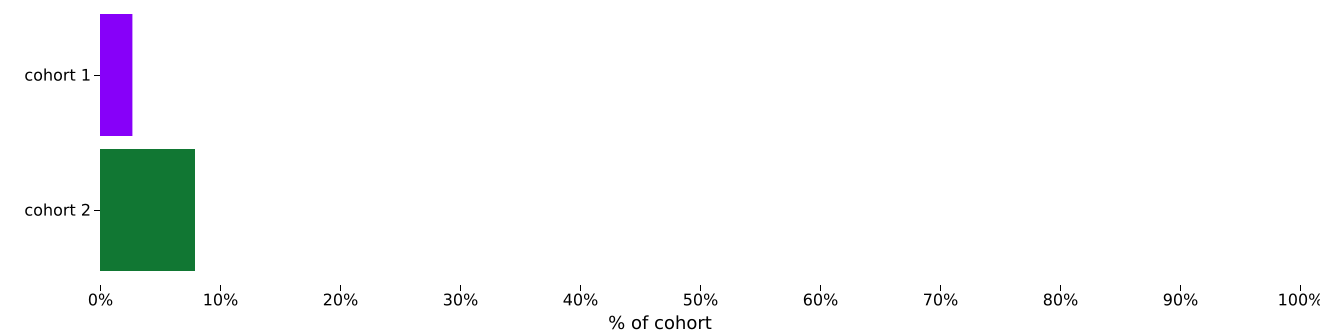 | | | | | | | |
|  | | **Kaplan - Meier survival analysis** | | | | | | | | | | |
|  | | |  | Cohort | | | Patients in cohort | Patients with outcome | Median survival (days) | Survival probability at end of time window | | |
|  | | |  | 1 | | KTRDM+GLP-1 RAs | 3,297 | 89 | -- | 95.22% | | |
|  | | |  | 2 | | KTRDM-GLP-1 RAs | 3,297 | 261 | -- | 88.17% | | |
|  | | | | | | | | | | | | |
|  | | |  |  | | | χ^2^ | df | p |  |  |  |
|  | | |  | **Log-Rank Test** | | | 61.559 | 1 | 0.000 |  |  |  |
|  | | | | | | | | | | | | |
|  | | |  |  | | | Hazard Ratio | 95% CI | χ^2^ | df | p | |
|  | | |  | **Hazard Ratio and Proportionality** | | | 0.394 | (0.310, 0.502) | 0.092 | 1 | 0.761 | |
|  | | | | | | | | | | | | |
|  | |  | | | 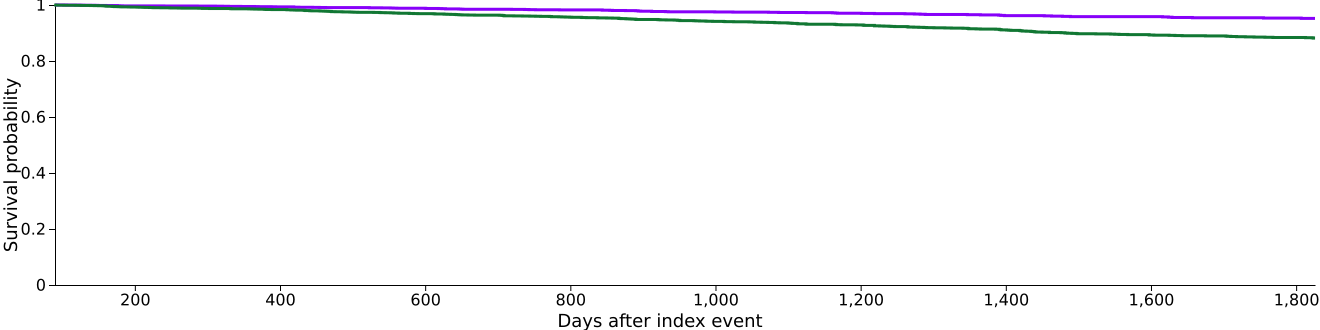 | | | | | | | |
|  | | **Number of instances** | | | | | | | | | | |
|  | | |  | Cohort | | | Patients in cohort | Patients with outcome | Mean | Standard Deviation | Median | |
|  | | |  | 1 | | KTRDM+GLP-1 RAs | 3,297 | 89 | 1.118 | 0.325 | 1 | |
|  | | |  | 2 | | KTRDM-GLP-1 RAs | 3,297 | 261 | 1.153 | 0.361 | 1 | |
|  | | | | | | | | | | | | |
|  | | |  |  | | | t | df | p |  |  |  |
|  | | |  | **Test Statistics** | | | -1.501 | 348 | 0.134 |  |  |  |
|  | | | | | | | | | | | | |
|  | |  | | | 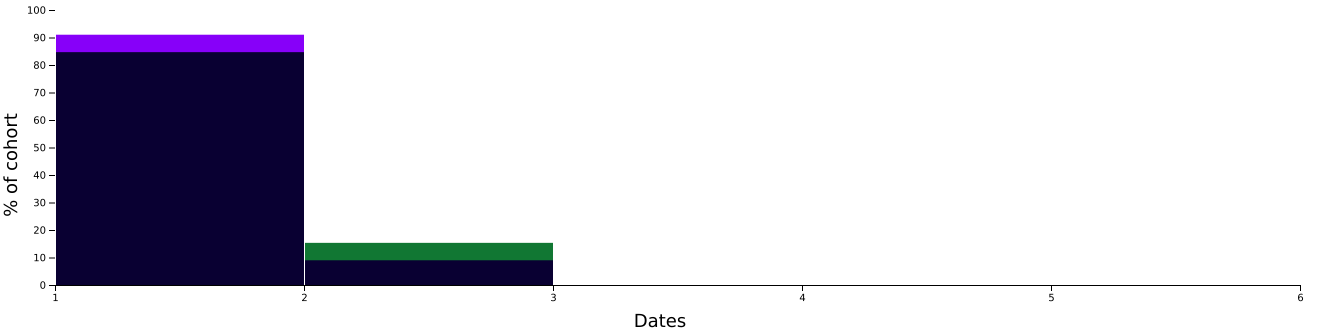 | | | | | | | |
| **Abbreviations:** GLP-1 RA; glucagon-like peptide-1 receptor agonist; KTR, kidney transplant recipient **Supplemental Table 5.** Risk of MACEs in KRTs with T2DM: Comparison between GLP-1 RAs users and non-users after propensity score matchingThe table presents the results from a cohort analysis after propensity score matching. Patients with a history of MACEs were included in the baseline cohort but excluded from this analysis. Cohort 1 consists of KTRs with T2DM who received GLP-1 RAs, while Cohort 2 includes those who did not use GLP-1 RAs. | | | | | | | | | | | | |
| **MACE** | | | | | | | | | | | | |
|  | | **Risk analysis excluding patients with outcome prior to the time window** | | | | | | | | | | |
|  |  | | | Cohort | | | Patients in cohort | Patients with outcome | Risk | | | |
|  | | |  | 1 | | KTRDM+GLP-1 RAs | 2,770 | 193 | 0.070 | | | |
|  | | |  | 2 | | KTRDM-GLP-1 RAs | 2,791 | 335 | 0.120 | | | |
|  | | | | | | | | | | | | |
|  | | |  |  | | |  | 95% CI | z | p |  |  |
|  | | |  | **Risk Difference** | | | -0.050 | (-0.066, -0.035) | -6.405 | 0.000 |  |  |
|  | | |  | **Risk Ratio** | | | 0.580 | (0.490, 0.687) | N/A | N/A |  |  |
|  | | |  | **Odds Ratio** | | | 0.549 | (0.456, 0.661) | N/A | N/A |  |  |
|  | | | | | | | | | | | | |
|  | |  | | | 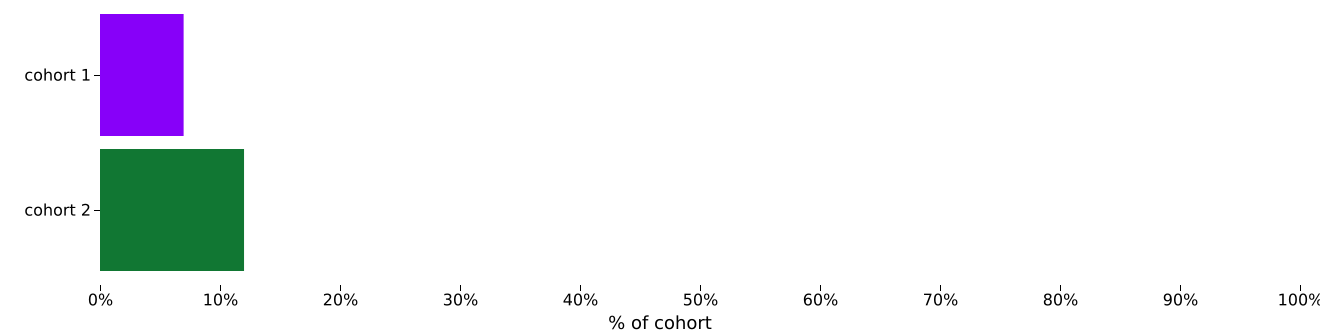 | | | | | | | |
|  | |  | | | 527 patients in Cohort 1 and 506 patients in Cohort 2 were excluded from results because they had the outcome prior to the time window. | | | | | | | |
|  | | **Kaplan - Meier survival analysis excluding patients with outcome prior to the time window** | | | | | | | | | | |
|  | | |  | Cohort | | | Patients in cohort | Patients with outcome | Median survival (days) | Survival probability at end of time window | | |
|  | | |  | 1 | | KTRDM+GLP-1 RAs | 2,770 | 193 | -- | 88.08% | | |
|  | | |  | 2 | | KTRDM-GLP-1 RAs | 2,791 | 335 | -- | 82.77% | | |
|  | | | | | | | | | | | | |
|  | | |  |  | | | χ^2^ | df | p |  |  |  |
|  | | |  | **Log-Rank Test** | | | 20.757 | 1 | 0.000 |  |  |  |
|  | | | | | | | | | | | | |
|  | | |  |  | | | Hazard Ratio | 95% CI | χ^2^ | df | p | |
|  | | |  | **Hazard Ratio and Proportionality** | | | 0.664 | (0.556, 0.793) | 0.054 | 1 | 0.816 | |
|  | | | | | | | | | | | | |
|  | |  | | | 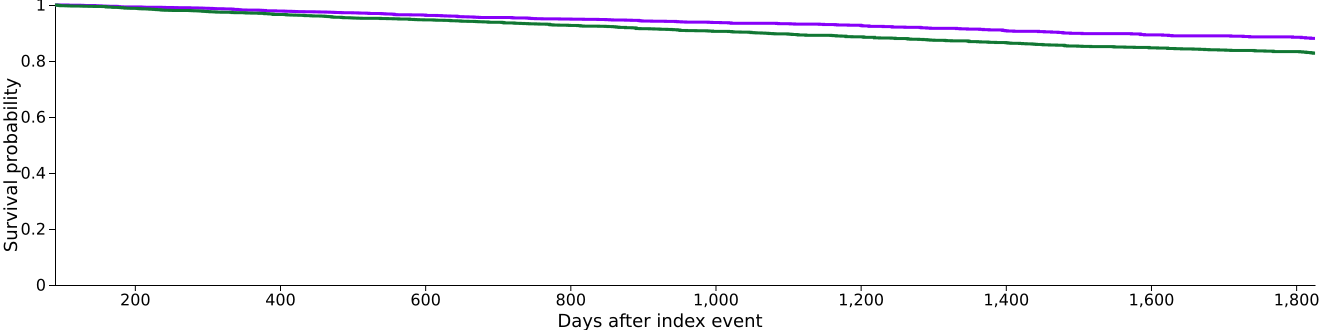 | | | | | | | |
|  | |  | | | 527 patients in Cohort 1 and 506 patients in Cohort 2 were excluded from results because they had the outcome prior to the time window. | | | | | | | |
|  | | **Number of instances excluding patients with outcome prior to the time window** | | | | | | | | | | |
|  | | |  | Cohort | | | Patients in cohort | Patients with outcome | Mean | Standard Deviation | Median | |
|  | | |  | 1 | | KTRDM+GLP-1 RAs | 2,770 | 193 | 4.803 | 10.768 | 1 | |
|  | | |  | 2 | | KTRDM-GLP-1 RAs | 2,791 | 335 | 3.164 | 6.436 | 1 | |
|  | | | | | | | | | | | | |
|  | | |  |  | | | t | df | p |  |  |  |
|  | | |  | **Test Statistics** | | | 2.189 | 526 | 0.029 |  |  |  |
|  | | | | | | | | | | | | |
|  | |  | | | 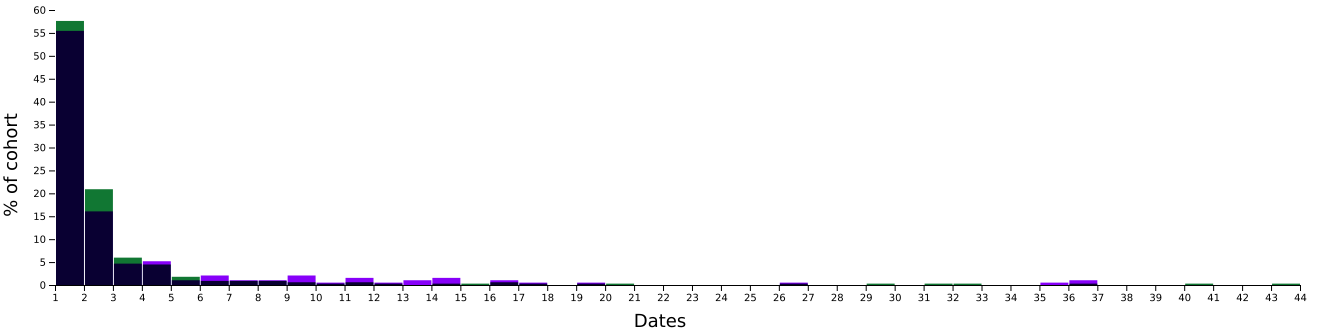 | | | | | | | |
| 4 data points for Cohort 1 and 2 data points for Cohort 2 were omitted for display purposes.  527 patients in Cohort 1 and 506 patients in Cohort 2 were excluded from results because they had the  outcome prior to the time window.  **Abbreviations:** GLP-1 RA; glucagon-like peptide-1 receptor agonist; KTR, kidney transplant recipient; MACE, major adverse cardiac event; N/A, not applicable **Supplemental Table 6.** Risk of MAKEs in KRTs with T2DM: Comparison between GLP-1 RAs users and non-users after propensity score matchingThe table presents the results from a cohort analysis after propensity score matching. Cohort 1 consists of KTRs with T2DM who received GLP-1 RAs, while Cohort 2 includes those who did not use GLP-1 RAs. | | | | | | | | | | | | |
| **MAKE** | | | | | | | | | | | | |
|  | | **Risk analysis** | | | | | | | | | | |
|  |  | | | Cohort | | | Patients in cohort | Patients with outcome | Risk | | | |
|  | | |  | 1 | | KTRDM+GLP-1 RAs | 3,297 | 407 | 0.123 | | | |
|  | | |  | 2 | | KTRDM-GLP-1 RAs | 3,297 | 667 | 0.202 | | | |
|  | | | | | | | | | | | | |
|  | | |  |  | | |  | 95% CI | z | p |  |  |
|  | | |  | **Risk Difference** | | | -0.079 | (-0.097, -0.061) | -8.671 | 0.000 |  |  |
|  | | |  | **Risk Ratio** | | | 0.610 | (0.545, 0.683) | N/A | N/A |  |  |
|  | | |  | **Odds Ratio** | | | 0.555 | (0.486, 0.635) | N/A | N/A |  |  |
|  | | | | | | | | | | | | |
|  | |  | | | 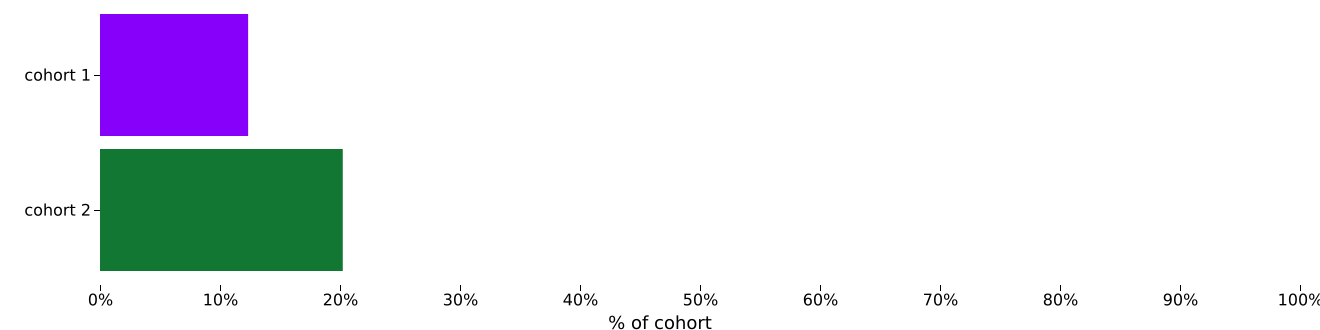 | | | | | | | |
|  | | **Kaplan - Meier survival analysis** | | | | | | | | | | |
|  | | |  | Cohort | | | Patients in cohort | Patients with outcome | Median survival (days) | Survival probability at end of time window | | |
|  | | |  | 1 | | KTRDM+GLP-1 RAs | 3,297 | 407 | -- | 81.29% | | |
|  | | |  | 2 | | KTRDM-GLP-1 RAs | 3,297 | 667 | -- | 72.98% | | |
|  | | | | | | | | | | | | |
|  | | |  |  | | | χ^2^ | df | p |  |  |  |
|  | | |  | **Log-Rank Test** | | | 44.412 | 1 | 0.000 |  |  |  |
|  | | | | | | | | | | | | |
|  | | |  |  | | | Hazard Ratio | 95% CI | χ^2^ | df | p | |
|  | | |  | **Hazard Ratio and Proportionality** | | | 0.659 | (0.583, 0.746) | 0.152 | 1 | 0.697 | |
|  | | | | | | | | | | | | |
|  | |  | | | 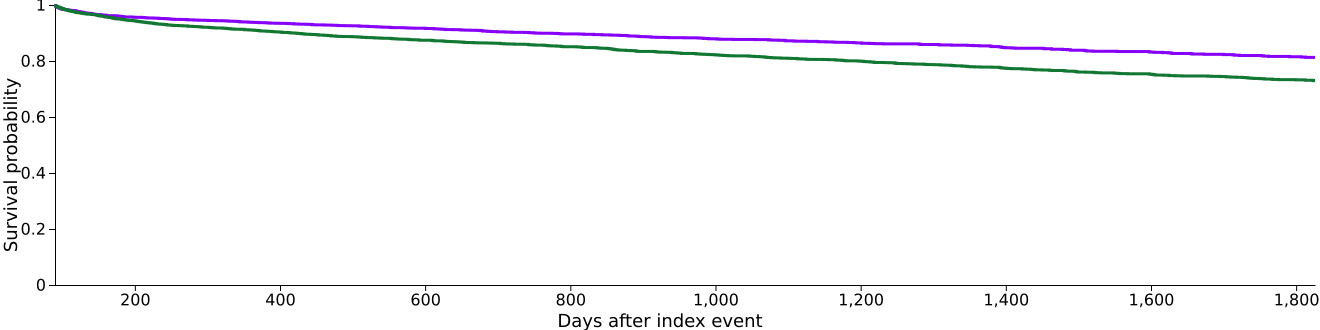 | | | | | | | |
|  | | **Number of instances** | | | | | | | | | | |
|  | | |  | Cohort | | | Patients in cohort | Patients with outcome | Mean | Standard Deviation | Median | |
|  | | |  | 1 | | KTRDM+GLP-1 RAs | 3,297 | 407 | 8.243 | 16.249 | 2 | |
|  | | |  | 2 | | KTRDM-GLP-1 RAs | 3,297 | 667 | 9.819 | 18.496 | 2 | |
|  | | | | | | | | | | | | |
|  | | |  |  | | | t | df | p |  |  |  |
|  | | |  | **Test Statistics** | | | -1.417 | 1072 | 0.157 |  |  |  |
|  | | | | | | | | | | | | |
|  | |  | | | 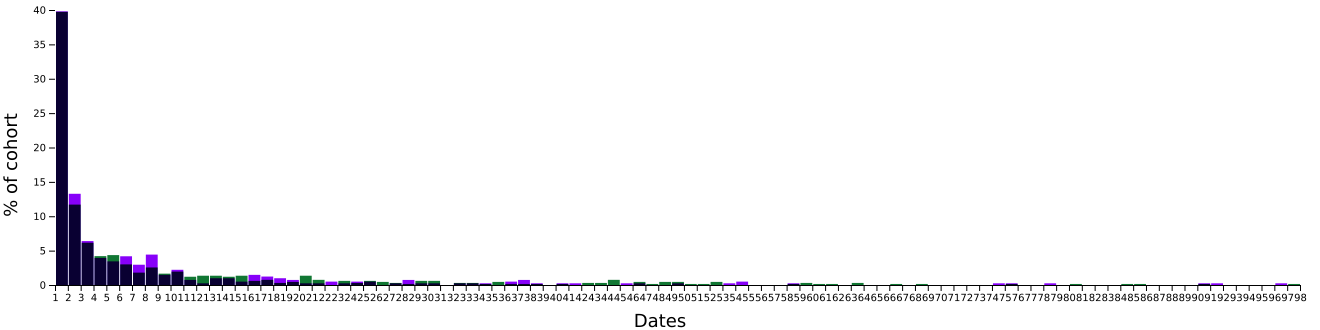 | | | | | | | |
|  | |  | | | 1 data points for Cohort 1 and 7 data points for Cohort 2 were omitted for display purposes. | | | | | | | |

**Abbreviations:** GLP-1 RA; glucagon-like peptide-1 receptor agonist; KTR, kidney transplant recipient; MAKE, major adverse kidney event; N/A, not applicable

**Supplemental Table 7.** Incidence rate ratios and E-values of outcomes of interest among the GLP-1 RAs users compared to the nonuser after propensity score matching

| Outcome | Total | Event | Mean follow up years | E-value (lower limit of CI) |
| --- | --- | --- | --- | --- |
| **All-cause mortality** | 6594 | 350 | 4.1 | 4.51 (3.40) |
| GLP-1RAs user | 3297 | 89 | 4.2 |  |
| GLP-1RAs non-user | 3297 | 261 | 4.0 |  |
| **MACE** | 5561 | 528 | 3.8 | 2.38 (1.83) |
| GLP-1RAs user | 2770 | 193 | 3.9 |  |
| GLP-1RAs non-user | 2791 | 355 | 3.7 |  |
| **MAKE** | 6594 | 1074 | 3.7 | 2.00 (1.75) |
| GLP-1RAs non-user | 3297 | 407 | 3.8 |  |
| GLP-1RAs non-user | 3297 | 667 | 3.5 |  |

**Abbreviations:** CI, confidence interval; GLP-1 RA; glucagon-like peptide-1 receptor agonist; MACE, major adverse cardiac event; MAKE, major adverse kidney events

**Supplemental Table 8.** Sensitivity analysis for all-cause mortality, MACEs and MAKEs between GLP-1 RAs user and GLP-1 RAs non-user

|  | **Mortality**  **aHR (95%CI)** | **MACE**  **aHR (95%CI)** | **MAKE**  **aHR (95%CI)** |  |
| --- | --- | --- | --- | --- |
| **All eligible subjects without weighting** | 0.29 (0.23-0.37) |  |  |  |
| 1:1 PSM, caliper=0.1 | 0.39 (0.31-0.50) | 0.66 (0.56-0.79) | 0.66 (0.58-0.75) |  |
| **Eligible subjects with different exclusion criteria, 1:1 PSM** |  |  |  | |
| Exclude patients received dialysis within 1  months post-transplant | 0.45 (0.35-0.57) | 0.61 (0.50-0.75) | 0.56 (0.48-0.65) |  |
| Exclude patients received dialysis within 2  months post-transplant | 0.49 (0.37-0.65) | 0.67 (0.54-0.83) | 0.60 (0.51-0.70) |  |
| Exclude patients received dialysis within 3  months post-transplant | 0.53 (0.39-0.72) | 0.62 (0.52-0.74) | 0.62 (0.52-0.74) |  |
| **Cox regression models with different covariates** |  |  |  |  |
| Model 1 (Original model except age) | 0.35 (0.27-0.46) | 0.56 (0.46-0.67) | 0.60 (0.53-0.69) |  |
| Model 2 (Original model except gender) | 0.38 (0.29-0.49) | 0.61 (0.51-0.73) | 0.63 (0.56-0.72) |  |
| Model 3 (Original model except ethnicity) | 0.32 (0.24-0.41) | 0.57 (0.47-0.69) | 0.60 (0.52-0.68) |  |
| Model 4 (Original model except medication) | 0.38 (0.30-0.49) | 0.63 (0.52-0.76) | 0.62 (0.55-0.70) |  |
|  |  |  |  |  |

**Abbreviations:** aHR, adjusted hazard ratio; CI, confidence interval; GLP-1 RA; glucagon-like peptide-1 receptor agonist; MACE, major adverse cardiac event; MAKE, major adverse kidney even; PSM, propensity score matching

**Supplemental Table 9.** Specificity analysis for all-cause mortality, MACEs and MAKEs between GLP-1 RAs user and GLP-1 RAs non-user

|  | **Mortality**  **aHR (95%CI)** | **MACE**  **aHR (95%CI)** | **MAKE**  **aHR (95%CI)** |  |
| --- | --- | --- | --- | --- |
| **Continued use versus non-use of GLP-1 RAs from 3 to 6 months post-transplant** | 0.30 (0.23-0.39) | 0.52 (0.43-0.63) | 0.57 (0.50-0.66) |  |
| **Timing of risk analyses** |  |  |  | |
| 1-year post-transplant | 0.35 (0.18-0.65) | 0.61 (0.39-0.94) | 0.68 (0.56-0.83) |  |
| 3-year post-transplant | 0.31 (0.22-0.45) | 0.64 (0.50-0.82) | 0.65 (0.56-0.76) |  |

**Abbreviations:** aHR, adjusted hazard ratio; CI, confidence interval; GLP-1 RA; glucagon-like peptide-1 receptor agonists; MACE, major adverse cardiac event; MAKE, major adverse kidney event

**Supplemental Table 10.** Specificity analysis for all-cause mortality, MACEs and MAKEs between patients receiving both GLP-1 RAs and SGLT2is versus those who did not receive either medication

|  |  | **aHR (95%CI)** | **P value** |
| --- | --- | --- | --- |
| **GLP-1 RAs+** **SGLT2is users versus GLP-1 RAs+ SGLT2is non-users** |  |  |  |
| **All-cause mortality** |  | 0.29 (0.16-0.53) | <0.001 |
| **MACE** |  | 0.52 (0.33-0.80) | <0.001 |
| **MAKE** |  | 0.52 (0.38-0.70) | <0.001 |

**Abbreviations:** aHR, adjusted hazard ratio; CI, confidence interval; GLP-1 RA; glucagon-like peptide-1 receptor agonists; MACE, major adverse cardiac event; MAKE, major adverse kidney event; SGLT2i, sodium-glucose cotransporter 2 inhibitor

**Supplemental Table 11.** Specificity analysis for all-cause mortality, MACEs and MAKEs between GLP-1 RAs user and patients using DPP-4is, TZDs or SUs

|  |  | **aHR (95%CI)** | **P value** |
| --- | --- | --- | --- |
| **GLP-1 RAs versus DPP4i/ SU/ TZD** |  |  |  |
| **All-cause mortality** |  | 0.49 (0.35 -0.68) | <0.001 |
| **MACE** |  | 0.70 (0.56-0.90) | 0.004 |
| **MAKE** |  | 0.68 (0.58-0.79) | <0.001 |

**Abbreviations:** aHR, adjusted hazard ratio; CI, confidence interval; DPP-4i, dipeptidyl peptidase-4 inhibitor; SU, sulfonylurea; TZD, thiazolidinedione; GLP-1 RA; glucagon-like peptide-1 receptor agonists; MACE, major adverse cardiac event; MAKE, major adverse kidney event

**Supplemental Table 12.** Comparative HbA1c, body weight, LDL, and SBP between GLP-1 RAs user and GLP-1 RAs non-user

| **Time** | **Groups** | | **P value** |
| --- | --- | --- | --- |
|  | **GLP-1 RAs user** | **GLP-1 RAs non-user** |  |
| **HbA1c, %** |  |  |  |
| Baseline | 7.3 ± 1.7 | 7.2 ± 1.7 | 0.046 |
| 90-180 days post-transplant | 7.4 ± 1.6 | 7.3 ± 1.6 | 0.040 |
| 180-270 days post-transplant | 7.5 ± 1.6 | 7.4 ± 1.7 | 0.072 |
| **Body weight** |  |  |  |
| Baseline | 200.0 ± 46.3 | 197.4 ± 46.2 | 0.023 |
| 90-180 days post-transplant | 199.6 ± 46.1 | 196.7 ± 47.6 | 0.743 |
| 180-270 days post-transplant | 200.9± 46.1 | 197.8 ± 48.0 | 0.093 |
| **LDL, mg/dL** |  |  |  |
| Baseline | 79.3 ± 34.1 | 80.6 ± 37.7 | 0.260 |
| 90-180 days post-transplant | 78.4 ± 32.0 | 78.8 ± 34.5 | 0.636 |
| 180-270 days post-transplant | 80.7 ± 34.4 | 83.7 ± 35.6 | 0.110 |
| **SBP, mmHg** |  |  |  |
| Baseline | 133.0 ± 21.7 | 133.0 ± 23.9 | 0.918 |
| 90-180 days post-transplant | 133.0 ± 19.0 | 132.4 ± 23.7 | 0.271 |
| 180-270 days post-transplant | 133.2± 19.0 | 133.8 ± 19.5 | 0.219 |

**Abbreviations**: GLP-1 RAs, glucagon-like peptide-1 receptor agonists; HbA1c, glycated hemoglobin; SBP systolic blood pressure

**Supplemental Table 13.** Landmark analysis for primary outcomes across different cohort selection period

|  | **aHR (95%CI)** | **P-value** |
| --- | --- | --- |
| **Usage of GLP-1RAs within 2 months post-transplant** |  |  |
| All-cause mortality | 0.37 (0.29,0.48) | <0.001 |
| MACE | 0.65 (0.54,0.78) | <0.001 |
| MAKE | 0.59 (0.52,0.66) | <0.001 |
| **Usage of GLP-1RAs within 6 months post-transplant** |  |  |
| All-cause mortality | 0.34 (0.26,0.44) | <0.001 |
| MACE | 0.61 (0.51,0.73) | <0.001 |
| MAKE | 0.62 (0.55,0.70) | <0.001 |
| **Usage of GLP-1RAs within 9 months post-transplant** |  |  |
| All-cause mortality | 0.38 (0.28,0.50) | <0.001 |
| MACE | 0.72 (0.60,0.87) | <0.001 |
| MAKE | 0.63 (0.55,0.72) | <0.001 |
| **Usage of GLP-1RAs within 12 months post-transplant** |  |  |
| All-cause mortality | 0.32 (0.23,0.43) | <0.001 |
| MACE | 0.64 (0.53,0.79) | <0.001 |
| MAKE | 0.66 (0.57,0.77) | <0.001 |

**Abbreviations**: aHR, adjusted hazard ratio; CI, confidence interval; GLP-1 RA; glucagon-like peptide-1 receptor agonist; MACE, major adverse cardiac event; MAKE, major adverse kidney event

**Supplemental Table 14.** Positive and negative exposure controls

|  | **aHR (95%CI)** | **P-value** |
| --- | --- | --- |
| **Usage of SGLT2is** |  |  |
| All-cause mortality | 0.44 (0.33, 0.60) | <0.001 |
| MACE | 0.68 (0.54, 0,85) | <0.001 |
| MAKE | 0.62 (0.53, 0.73) | <0.001 |
| **Usage of topical urea** |  |  |
| All-cause mortality | 1.07 (0.65, 1.78) | 0.792 |
| MACE | 1.19 (0.75, 1.90) | 0.471 |
| MAKE | 0.88 (0.61, 1.6) | 0.434 |

**Abbreviations:** aHR, adjusted hazard ratio; CI, confidence interval; SGLT2i, sodium-glucose cotransporter 2 inhibitor

1. Supplementary figure

**Supplemental Figure 1. Graphic abstract**

**
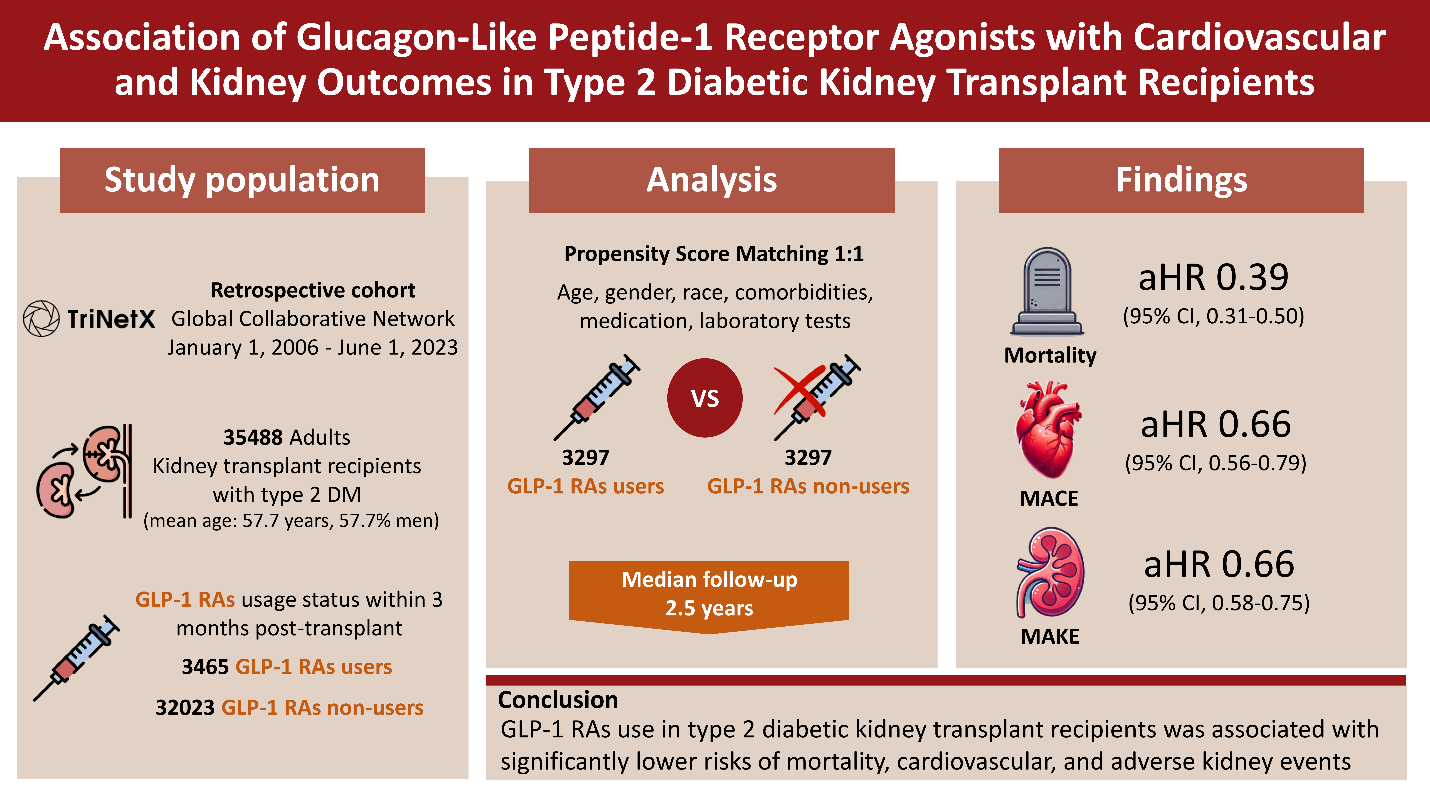
**

1. **STROBE Statement—checklist of items that should be included in reports of observational studies**

|  | | Item No | Recommendation | Page No |  |
| --- | --- | --- | --- | --- | --- |
| **Title and abstract** | | 1 | (*a*) Indicate the study’s design with a commonly used term in the title or the abstract | P.1 |  |
|  |  |  | (*b*) Provide in the abstract an informative and balanced summary of what was done and what was found | P.3-4 |  |
| Introduction | | | |  |  |
| Background/rationale | | 2 | Explain the scientific background and rationale for the investigation being reported | P.5-6 |  |
| Objectives | | 3 | State specific objectives, including any prespecified hypotheses | P.6 |  |
| Methods | | | |  |  |
| Study design | | 4 | Present key elements of study design early in the paper | P.7-9 |  |
| Setting | | 5 | Describe the setting, locations, and relevant dates, including periods of recruitment, exposure, follow-up, and data collection | P.7-9 |  |
| Participants | | 6 | (*a*) *Cohort study*—Give the eligibility criteria, and the sources and methods of selection of participants. Describe methods of follow-up  *Case-control study*—Give the eligibility criteria, and the sources and methods of case ascertainment and control selection. Give the rationale for the choice of cases and controls  *Cross-sectional study*—Give the eligibility criteria, and the sources and methods of selection of participants | P.8-9  Figure.1 |  |
|  |  |  | (*b*) *Cohort study*—For matched studies, give matching criteria and number of exposed and unexposed  *Case-control study*—For matched studies, give matching criteria and the number of controls per case | P.8-9  Figure.1 |  |
| Variables | | 7 | Clearly define all outcomes, exposures, predictors, potential confounders, and effect modifiers. Give diagnostic criteria, if applicable | P.9 |  |
| Data sources/ measurement | | 8* | For each variable of interest, give sources of data and details of methods of assessment (measurement). Describe comparability of assessment methods if there is more than one group | P.6-7 |  |
| Bias | | 9 | Describe any efforts to address potential sources of bias | P.8-10 |  |
| Study size | | 10 | Explain how the study size was arrived at | Figure 1 |  |
| Quantitative variables | | 11 | Explain how quantitative variables were handled in the analyses. If applicable, describe which groupings were chosen and why | P.7 |  |
| Statistical methods | | 12 | (*a*) Describe all statistical methods, including those used to control for confounding | P.11-12 |  |
|  |  |  | (*b*) Describe any methods used to examine subgroups and interactions | P.9-10 |  |
|  |  |  | (*c*) Explain how missing data were addressed | P.11 |  |
|  |  |  | (*d*) *Cohort study*—If applicable, explain how loss to follow-up was addressed  *Case-control study*—If applicable, explain how matching of cases and controls was addressed  *Cross-sectional study*—If applicable, describe analytical methods taking account of sampling strategy | P.11 |  |
|  |  |  | (*e*) Describe any sensitivity analyses | P.9-10 |  |
| Results | | | |  | |
| Participants | 13* | (a) Report numbers of individuals at each stage of study—eg numbers potentially eligible, examined for eligibility, confirmed eligible, included in the study, completing follow-up, and analysed | | P.12-13  Figure 1 | |
|  |  | (b) Give reasons for non-participation at each stage | | P.12-13  Figure 1 | |
|  |  | (c) Consider use of a flow diagram | | Figure 1 | |
| Descriptive data | 14* | (a) Give characteristics of study participants (eg demographic, clinical, social) and information on exposures and potential confounders | | P.12  Table 1 | |
|  |  | (b) Indicate number of participants with missing data for each variable of interest | | P.12  Table S3 | |
|  |  | (c) *Cohort study*—Summarise follow-up time (eg, average and total amount) | | P.12  Table S7 | |
| Outcome data | 15* | *Cohort study*—Report numbers of outcome events or summary measures over time  *Case-control study—*Report numbers in each exposure category, or summary measures of exposure  *Cross-sectional study—*Report numbers of outcome events or summary measures | | P.12-13 | |
| Main results | 16 | (*a*) Give unadjusted estimates and, if applicable, confounder-adjusted estimates and their precision (eg, 95% confidence interval). Make clear which confounders were adjusted for and why they were included | | Table S8 | |
|  |  | (*b*) Report category boundaries when continuous variables were categorized | | Table 1 | |
|  |  | (*c*) If relevant, consider translating estimates of relative risk into absolute risk for a meaningful time period | | P.12-13 | |
| Other analyses | 17 | Report other analyses done—eg analyses of subgroups and interactions, and sensitivity analyses | | P.13  Figure 4 Table S8 | |
| Discussion | | | |  | |
| Key results | 18 | Summarise key results with reference to study objectives | | P.15-16 | |
| Limitations | 19 | Discuss limitations of the study, taking into account sources of potential bias or imprecision. Discuss both direction and magnitude of any potential bias | | P.17-18 | |
| Interpretation | 20 | Give a cautious overall interpretation of results considering objectives, limitations, multiplicity of analyses, results from similar studies, and other relevant evidence | | p.19 | |
| Generalisability | 21 | Discuss the generalisability (external validity) of the study results | | N/A | |
| Other information | | | |  | |
| Funding | 22 | Give the source of funding and the role of the funders for the present study and, if applicable, for the original study on which the present article is based | | P.20 | |

*Give information separately for cases and controls in case-control studies and, if applicable, for exposed and unexposed groups in cohort and cross-sectional studies.

**Note:** An Explanation and Elaboration article discusses each checklist item and gives methodological background and published examples of transparent reporting. The STROBE checklist is best used in conjunction with this article (freely available on the Web sites of PLoS Medicine at http://www.plosmedicine.org/, Annals of Internal Medicine at http://www.annals.org/, and Epidemiology at http://www.epidem.com/). Information on the STROBE Initiative is available at www.strobe-statement.org.
